# Mathematical Methods for Measuring the Visually Enhanced Vestibulo–Ocular Reflex and Preliminary Results from Healthy Subjects and Patient Groups

**DOI:** 10.3389/fneur.2018.00069

**Published:** 2018-02-12

**Authors:** Jorge Rey-Martinez, Angel Batuecas-Caletrio, Eusebi Matiño, Gabriel Trinidad-Ruiz, Xabier Altuna, Nicolas Perez-Fernandez

**Affiliations:** ^1^Otorhinolaringology, Hospital Universitario Donostia, San Sebastian, Spain; ^2^Complejo Hospitalario de Salamanca, Salamanca, Spain; ^3^Hospital General de Catalunya, Barcelona, Spain; ^4^University Hospital of Badajoz, Badajoz, Spain; ^5^Clínica Universidad de Navarra, Madrid, Spain

**Keywords:** Visually enhanced VOR, visual–vestibular interaction, CANVAS, vestibular schwannoma, gain, dessaccade, vestibulo–ocular reflex, video head impulse test, algorithms

## Abstract

**Background:**

Visually enhanced vestibulo–ocular reflex (VVOR) is a well-known bedside clinical test to evaluate visuo–vestibular interaction, with clinical applications in patients with neurological and vestibular dysfunctions. Owing to recently developed diagnostic technologies, the possibility to perform an easy and objective measurement of the VVOR has increased, but there is a lack of computational methods designed to obtain an objective VVOR measurement.

**Objectives:**

To develop a method for the assessment of the VVOR to obtain a gain value that compares head and eye velocities and to test this method in patients and healthy subjects.

**Methods:**

Two computational methods were developed to measure the VVOR test responses: the first method was based on the area under curve of head and eye velocity plots and the second method was based on the slope of the linear regression obtained for head and eye velocity data. VVOR gain and vestibulo–ocular reflex (VOR) gain were analyzed with the data obtained from 35 subjects divided into four groups: healthy (*N* = 10), unilateral vestibular with vestibular neurectomy (*N* = 8), bilateral vestibulopathy (*N* = 12), and cerebellar ataxia, neuropathy, and vestibular areflexia syndrome (CANVAS) (*N* = 5).

**Results:**

Intra-class correlation index for the two developed VVOR analysis methods was 0.99. Statistical differences were obtained by analysis of variance statistical method, comparing the healthy group (VVOR mean gain of 1 ± 0) with all other groups. The CANVAS group exhibited (VVOR mean gain of 0.4 ± 0.1) differences when compared to all other groups. VVOR mean gain for the vestibular bilateral group was 0.8 ± 0.1. VVOR mean gain in the unilateral group was 0.6 ± 0.1, with a Pearson’s correlation of 0.52 obtained when VVOR gain was compared to the VOR gain of the operated side.

**Conclusion:**

Two computational methods to measure the gain of VVOR were successfully developed. The VVOR gain values appear to objectively characterize the VVOR alteration observed in CANVAS patients, and also distinguish between healthy subjects and patients with some vestibular disorders.

## Introduction

In the experimental paradigm where a subject oscillates the head while viewing a static target in a stationary scene, a combination of the vestibulo–ocular reflex (VOR), smooth pursuit (SP), fixation, and the optokinetic reflex (OKN) produces almost perfectly compensatory eye movements to keep the visual target centered and clear (1). The neurological integration of the visual and vestibular inputs, and their associated reflexes, defines the visuo–vestibular interaction (VVI), which is fundamental not only to the process of dynamic visual acuity (2) but also spatial awareness and the ability to distinguish between world- and self-motion (3).

The visually enhanced VOR (VVOR) can be used at the bedside by asking the patient to fixate on the examiner’s nose while performing a continuous slow side-to-side oscillation. In a healthy subject, the eyes remain still, without significant refixation saccades. However, at extreme lateral head positions, some gaze-evoked nystagmus could make examination difficult. In cases of a hypoactive VOR, the eyes move away from the target in the direction of head rotation, leading to corrective “catch up” saccades back to the target (4). In the case of an abnormally high VOR gain, backup saccades will move the eye in the direction of head rotation (4). The response not only depends on the severity of the vestibular deficit but is also frequency-specific (5). Bedside testing has offered insights for the evaluation of mixed damage, as seen in cerebellar ataxia, neuropathy, and vestibular areflexia syndrome (CANVAS). In this case, the abnormal VVOR, when performed by an experienced examiner, reflects a deficit of the VOR, OKN, and SP (1, 6, 7).

Owing to the characteristics of the systems involved, the possibility of combining different frequencies of oscillatory head movement with earth-fixed visual stimulation have made this assessment very valuable in laboratory testing. At low to mid frequencies of stimulation, the OKN contributes to eye stabilization, but for high frequencies, the vestibulo–ocular reflex (VOR) overrules the OKN (8). During sinusoidal stimulation, the SP deteriorates when target frequency increases, while keeping the maximum amplitude of excursion constant (9). When following targets with constant velocity, it has been shown that the gain deteriorates once target velocity approaches 100°/s (1). Using low frequency stimulation, the combination of OKN and VOR during VVI testing has offered insight to the differential diagnosis of central versus peripheral disorders (10, 11). High-frequency oscillations have usually been performed with special equipment while rotating the entire subject but also just the head, this is known as the head-only rotational test.

In recent years, new clinical devices have been designed for measurement of the VOR and VVOR based on head-mounted goggles with built-in high-speed cameras and gyroscopes. This test is also known as the video head impulse test (vHIT), originally designed to assist and enhance the interpretation of the beside head impulse test (12). The new devices have provided a strong, universal, and easy method of measuring head and eye motion (13, 14, 15), and have expanded the ability to test head-only rotational VVOR at mid-frequency oscillations (1–2 Hz) at high peak velocities (200°/s).

The VVOR testing paradigm has a notable limitation; as with some of the commercially available systems, there is no objective, numerically measured result available for the VVOR test. This limits the results merely to the examiner’s impression of the eye and head velocity plots obtained.

The first aim of this study is to develop a method for the assessment of the VVOR test that provides a value for the comparison of head and eye velocities, and interposing saccades. The second aim is to assess the utility of this method by testing it on different groups of patients and healthy subjects.

## Materials and Methods

### The VVOR Test

For the VVOR test, the ICS Impulse^®^ ver. 4.0 (Otometrics A/S, Taastrup, Denmark) system was used. The subject was comfortably seated, looking straight ahead at a fixation point placed 1 m in front, at a height matching that of the subject’s eyes. After calibration, the test was initiated in a passive mode. This involved the examiner placing his/her hands on top of the subject’s head, and turning the subject’s head continuously from one side to the other for at least 20 s. The aims of this procedure were to: (1) maintain smooth movement; (2) prevent stopping at extreme head positions; (3) track the performance online to ensure a close match to a sinusoidal oscillation; (4) avoid left- or rightward bias during oscillations; (5) perform oscillations at 1–2 Hz, describing a symmetric arch of 40–50° of amplitude, 20–25° toward left side, and 20–25° toward right side; (6) obtain a maximum head velocity of 150–200°/s; and (7) perform 1–2 trials before performing the definitive test, which is registered for analysis.

### Development of VVOR Analysis Method

The developed method was written in universal script files for MATLAB (MATLAB Release 2015b, The MathWorks, Inc., Natick, MA, USA) and Octave (GNU Octave Release 4.0.3, John W. Eaton, David Bateman, Soren Hauberg, Rik Wehbring, www.octave.org).

**Figure 1:**
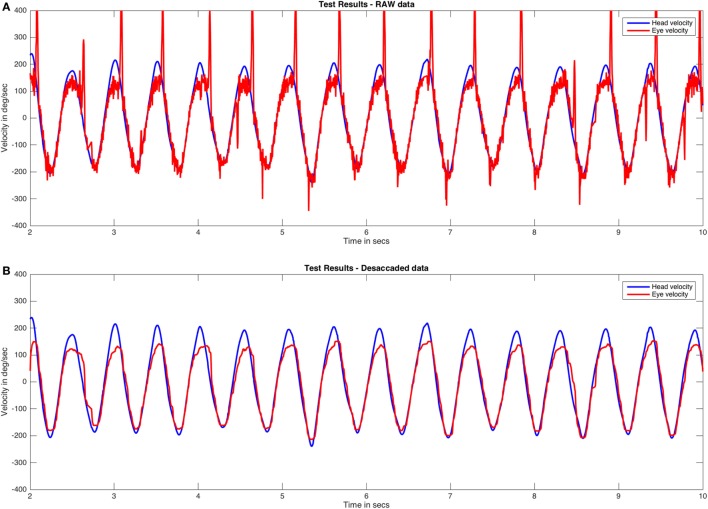
RAW and desaccaded visually enhanced VOR (VVOR) plots. **(A)** Original (RAW) VVOR plot from a patient with a unilateral vestibular hypofunction as a consequence of a retrolabyrinthine left vestibular nerve section performed due to a vestibular schwannoma. **(B)** Desaccaded eye curve and plot. Eye velocity plots were inverted to visually match the head velocity plots.

The original data were initially exported to a comma separated values (CSV) file format. The first MATLAB/Octave scripting task was to write a script to read the CSV file using a line-by-line reading method. The script stored the time stamp, eye velocity, and head velocity for the last recorded VVOR test in the file. A plot of the obtained data was generated to allow a quick overview of the original (RAW) data (Figure 1A).

While performing numerical analysis of the head and eye responses in the VVOR test, two main stages could introduce errors in calculation during a graphical- or fast Fourier transform-based analysis. The first issue was that the movement generated by the examiner was not as strictly periodic as that achieved using rotatory chair systems. This issue was resolved using online observation and performance of test trials before definitive evaluation and registration. The examiners involved in this study also had significant experience in performing vHIT and VVOR testing. The second issue was the frequent interruption of the smooth eye response due to the appearance of the fast phase of nystagmus (Figure 1A). To resolve this, the eye response was processed and “desaccaded” with a one-dimension median filter using the “medfilt1” MATLAB/Octave signal processing function with a window length parameter value of 30. The median filter function is defined by Pratt (16).

$$y(i)=\tilde{x}\left(i-\frac{n}{2}:i+\left(\frac{n}{2}-1\right)\right),$$

where *y* is the output vector, *x* is the input vector, *i* is the current function position, and *n* is the window length parameter. We selected the above window length parameter value after many trial-and-error attempts with real VVOR plots. Values that are too low retain most of the saccades on the output plot, while values that are too high flatten the eye response baseline. The processed VVOR with desaccaded data was then plotted (Figure 1B).

**Figure 2:**
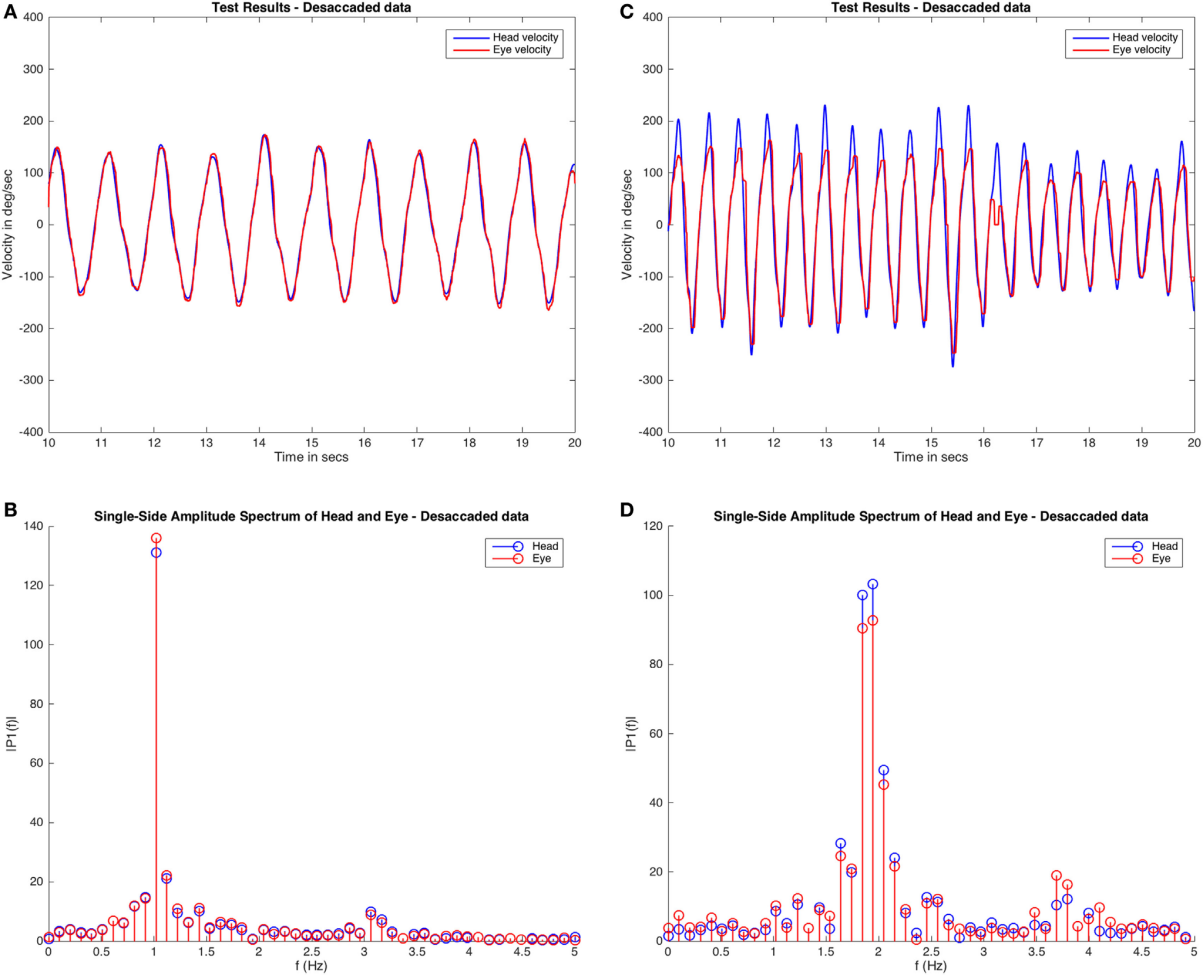
Fast Fourier transform of visually enhanced VOR (VVOR) results. The desaccaded plot and fast Fourier transform method assessment of two different subjects are shown. Panels **(A,B)** are plots obtained from the same healthy subject, and **(C,D)** are plots obtained from a patient with left vestibular hypofunction. These plots demonstrate some of the variabilities observed while testing. In the case of the subject shown in panel **(A)**, the VVOR test was performed with very symmetric head movements and, the amplitudes obtained in fast Fourier transform plot are mainly centered around a frequency of ~1.0 Hz. In contrast, for the patient [shown in **(B)**] the test appears more asymmetric in terms of head velocity and the values for head and eye movements obtained from fast Fourier transform are distributed at different frequencies, mainly at ~2.0 and ~1.8 Hz, with other significant amplitudes also registered at ~2.2 and ~1.6 Hz frequencies. Note also that for the second subject on the fast Fourier transform **(D)** the eye amplitudes (in red) always have a lower value than the head amplitudes (in blue), contrary to the normal subject **(B)** where both amplitudes are similar.

Next, a fast Fourier transform was applied to both the RAW and desaccaded VVOR data. The fast Fourier transform is a useful tool to graphically evaluate the periodicity of movements of the head during the VVOR test and also to evaluate the main frequency of the head movement (Figure 2).

To comply with the study aims, two methods were developed from the VVOR desaccaded data: (1) based on an area under the curve (AUC) calculation and (2) the eye versus head scatter plot (SCP).

**Figure 3:**
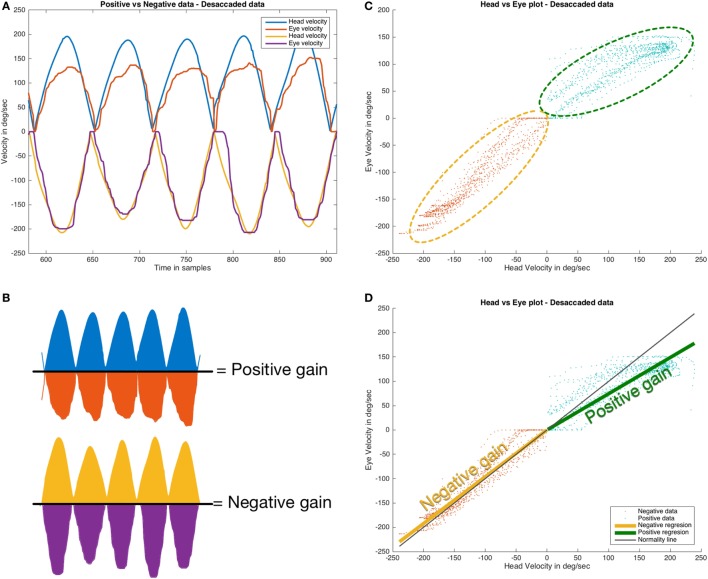
Methods for visually enhanced VOR gain calculation. **(A,B)** area under the curve; **(C,D)** eye versus head scatter plot. In the area under curve method, the data are split into rightward (positive) and leftward (negative) data **(A)** and then the area under the curve describing the eye movement was divided by the area under the curve describing the head movement for both rightward and leftward plots, providing the positive area under curve positive value (G_AUCp_) and negative area under curve value (G_AUCn_) as output **(B)**. In the second method, positive (rightward) and negative (leftward) head movement were plotted versus eye movement **(C)**. In the next step **(D)**, the data from the plots for positive and negative movement were analyzed with by linear regression; the slope of the regression line for rightward and leftward head movements was used to calculate the values of positive (G_SPp_) and negative (G_SPn_) gains, respectively. Eye velocity plots were inverted to visually match the head velocity plots.

For the AUC method, the first step was to split the positive and negative data. This was computed to obtain the AUC value using the MATLAB/Octave “trapz” function. A positive VVOR gain (G_AUCp_) is the AUC of eye and head velocities during rightward displacement of the head. A negative VVOR gain (G_AUCn_) is similarly obtained during the leftward displacement of the head (Figure 3A,B). We assumed “*a priori*” that leftward and rightward directions, respectively, match with negative and positive head velocity values given as output by the vHIT device, but this information was not provided by the manufacturer of the system used for the VVOR test (ICS impulse^®^ ver. 4.0 user’s guide and technical specifications).

In the SCP method, the desaccaded data were also analyzed to split the data: for positive and negative movement. Linear regression was computed using the “mldivide” (also denoted as “\”) function. This function computes the slope of the solved linear equation involving head and eye velocities. Positive and negative gains (respectively, G_SPp_ and G_SPn_) were obtained for head movements to the right and left side, respectively (Figure 3C,D).

As a corollary of this approach, the RAW VVOR eye data were subtracted from the desaccaded VVOR eye data, generating the eye saccade data. Saccades were computed using the “findpeaks” MATLAB/Octave function. The identified saccades were computed to calculate the total number of saccades and mean number of saccades per second for each test. A fast Fourier transform was performed to obtain head frequency stimulation, based on the maximum amplitude frequency value. Using the saccadic analysis data and fast Fourier transform data, the mean number of saccades per cycle was obtained. Other parameters, such as the peak head and eye velocities, were also computed.

The VVOR analysis methods described here have been published in their entirety as an open source script on the GitHub web repository: https://github.com/bendermh/VVOR and also as supplementary material of this manuscript.

### Clinical Application of VVOR Analysis Method

To investigate the clinical application of the developed method, we performed a multi-center prospective non-randomized study. For this study, 35 adult participants were consecutively recruited from four otolaryngology clinics between January 2016 and January 2017. Four senior experienced neuro-otologists examined the participants and unanimously agreed with the conditions and methodology of the VVOR testing. A vHIT was performed to measure the vestibulo–ocular reflex gain and identify refixation saccades (17).

Participants were divided into four groups. The “healthy” group included 10 subjects with no previous history of vestibular, oculomotor, or neurologic problems, and with good hearing and visual acuity. The second group consisted of 12 patients with peripheral bilateral vestibulopathy, all of whom had a VOR gain for the 6 semicircular canals, that was evaluated to be lower than the expected value, according to their respective ages (18, 19). The patients did not have any concomitant visual or neurological problems. The main diagnoses were bilateral Ménière’s disease, bilateral labyrinthitis, and bilateral chronic otitis media. The third group consisted of eight patients with unilateral vestibular hypofunction. All the patients were treated for unilateral vestibular schwannoma with retrolabyrinthine or translabyrinthine vestibular surgery at least 1 year before participating in this study. None of the patients had a history of any other ophthalmological or neurological disorder. The fourth group consisted of five patients with CANVAS who met the criteria for probable (one patient) or definite (four patients) diagnosis (20).

Written informed consent was obtained from all the patients. Since no novel or exceptional interventions were performed in this observational clinical study, only the approval of the local ethical committee for the corresponding institutions was required for the researchers. The study was designed and performed in accordance with the ethical guidelines of the 1975 Declaration of Helsinki.

### Statistics

Statistical analysis was performed using PSPP (GNU PSPP version 0.10.2, Plaff B., Free Software Foundation. Boston, MA, USA) and MATLAB, with a statistical toolbox.

The VVOR gain (both for the AUC and slope of the scatter plot methods), VVOR test frequency, and number of saccades were the main variables analyzed to characterize the measured VVOR response in the four participating groups. A time window of 10 s was defined as the optimal size to be analyzed for each participant. For all the groups, the left and right VOR gain measured during the vHIT was also collected.

The main variables were calculated using descriptive statistics. The Kolmogorov–Smirnov test was performed on all variables to assess their normality. For both VVOR gain measurement methods, concordance was assessed using the intra-class correlation coefficient. A value of ≥0.90 implied excellent concordance (21, 22). In addition, a Bland–Altman plot (23) was generated.

#### Intra-Group Tests

For each group, a *t*-test was performed to detect differences between positive and negative VVOR gain, and left and right VOR gain. In the CANVAS and unilateral vestibular groups, owing to the small sample sizes (*N* < 10), the Wilcoxon test was used to analyze the same intra-group variables.

Pearson’s correlation and linear regression were used to detect a possible relationship between VVOR and VOR gain values in the unilateral vestibular group (24). Positive and negative VVOR gain values were paired with operated and non-operated VOR gain values. Two correlations were considered: first, that the VVOR and VOR directions of head movement were the same (positive, rightward VVOR matches with right side VOR and, negative, leftward VVOR matches with left side VOR) and, second, that the VVOR direction was contrary to that of the VOR (positive direction VVOR matches with left side VOR, and negative VVOR matches with right side VOR).

#### Inter-Group Tests

A *t*-test was performed to compare the positive and negative VVOR gain values between the healthy group and all the other groups combined. To analyze the differences in VVOR positive and negative gain values between all groups separately, one-factor analysis of variance (ANOVA) with Bonferroni *post hoc* test was performed.

A *t*-test was used to detect differences in the number of saccades during the VVOR test and VVOR test frequency between each group and all other groups combined.

## Results

### VVOR Test and Application of the Developed Analysis Methods

The VVOR clinical test was successfully performed on all participants without any incident or difficulty. Trials were performed in all subjects/patients prior to the registration of the definitive tests. The developed methodologies (computer script files) were successfully executed without any run-time errors or warnings in all 35 cases, using both MATLAB and Octave environments.

**Figure 4:**
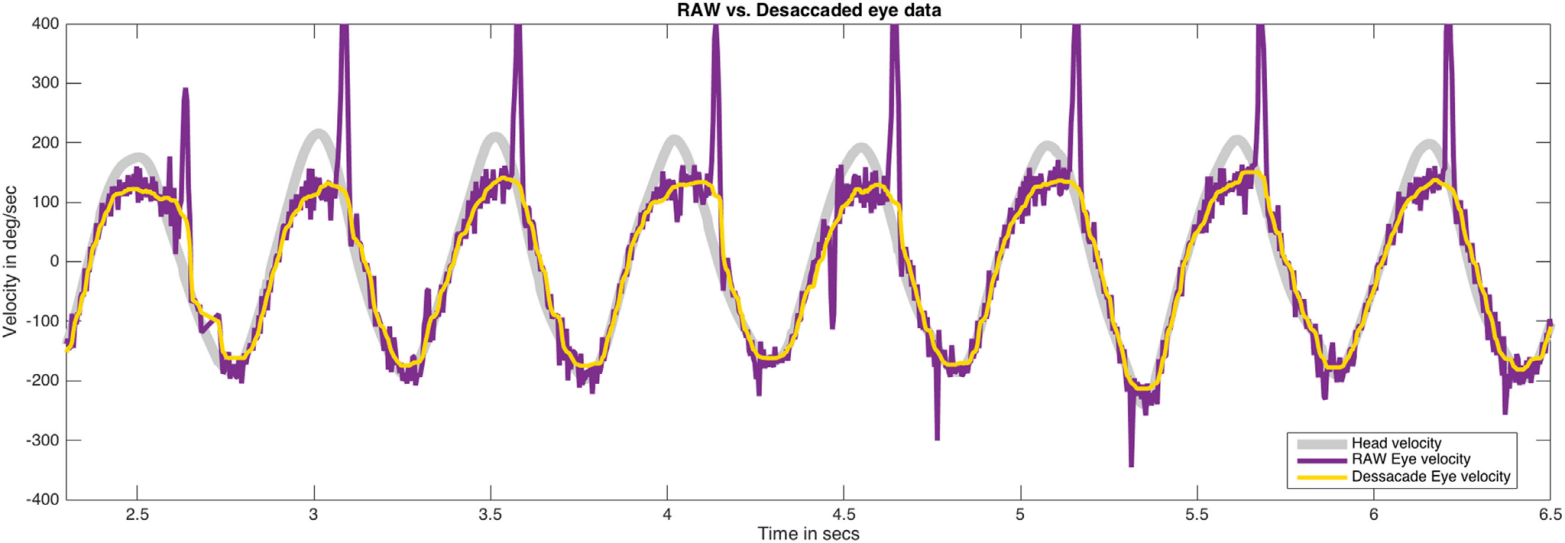
The effect of median filter processing on the plots obtained for saccadic eye movements. The plots obtained for head movement, RAW eye movement (with saccades), and desaccaded eye movement (without saccades) after applying median filter have been shown. This analysis method was carefully applied to determine the effect of filtering process in our data. No significant artifacts on eye data were found after the use of the median filter. Eye velocity plots were inverted to visually match the head velocity plots.

We analyzed the effects of the median filter used on different pathological records and did not find any evidence of signal alteration in the data during the process of saccadic movement removal (Figure 4). To allow the detection of artifacts caused by the median filter, the RAW data are always plotted in our analysis method. The head impulses performed in this study had a mean velocity of 109.08 ± 33.89°/s and a frequency of rotation of 1.37 ± 0.47 Hz.

### Patients

In Table 1, we present a summary of the subjects’ data and in Table 2, the different values of the variables analyzed. In both cases, the results are given for each group of subjects and patients.

**Table 1:** Descriptive characteristics of the healthy subjects and patients who participated in this study.

Parameters	Healthy	Vestibular bilateral	Vestibular unilateral	CANVAS
*N*	10	12	8	5
Sex ratio (male:female)	4:6	7:5	4:4	3:2
Mean age (min–max)	48 (27–76)	78 (58–91)	51 (30–70)	70 (54–86)
vHIT mean gain of vestibulo–ocular reflex	0.98 ± 0.04	0.43 ± 0.21	0.44 ± 0.11^a^	0.35 ± 0.26
			0.79 ± 0.1^b^	

*N, number of subjects; CANVAS, cerebellar ataxia, neuropathy, and vestibular areflexia syndrome; vHIT, video head impulse test*.

*^a^ represents vHIT gain on operated side*.

*^b^ represents vHIT gain on non-operated side*.

**Table 2:** Main values for measured visually enhanced VOR variables.

Method	Value	Healthy	Vestibular bilateral	Vestibular unilateral	CANVAS
FFT	Frequency (Hz)	1.52 ± 0.32	1 ± 0.48	1.76 ± 0.1	1.35 ± 0.51
Maximum	Head Vel. (°/s)	111.3 ± 36.2	88.3 ± 29.36	138.1 ± 29.65	106.8 ± 36.9
AUC	G_AUCp_	1.00 ± 0.03	0.84 ± 0.13	0.81 ± 0.12	0.45 ± 0.11
	G_AUCn_	1.00 ± 0.05	0.82 ± 0.15	0.73 ± 0.2	0.4 ± 0.12
SPC	G_SPp_	0.99 ± 0.03	0.84 ± 0.14	0.81 ± 0.12	0.43 ± 0.11
	G_SPn_	0.99 ± 0.03	0.83 ± 0.13	0.72 ± 0.21	0.41 ± 0.12
Saccades	Saccades/cycle	0 ± 0	3 ± 1.62	2.12 ± 0.85	4.08 ± 2.12

*FFT, fast Fourier Transform method; Head Vel., maximum head velocity; AUC, area under curve method; SPC, slope of scatter plot regression method; G_AUCp_, positive gain value obtained with area under curve method; G_AUCn_, negative gain value obtained with area under curve method; G_SPp_, positive gain value obtained with slope of scatter plot regression method; G_SPn_, negative gain value obtained with slope of scatter plot regression method; CANVAS, cerebellar ataxia, neuropathy, and vestibular areflexia syndrome*.

**Figure 5:**
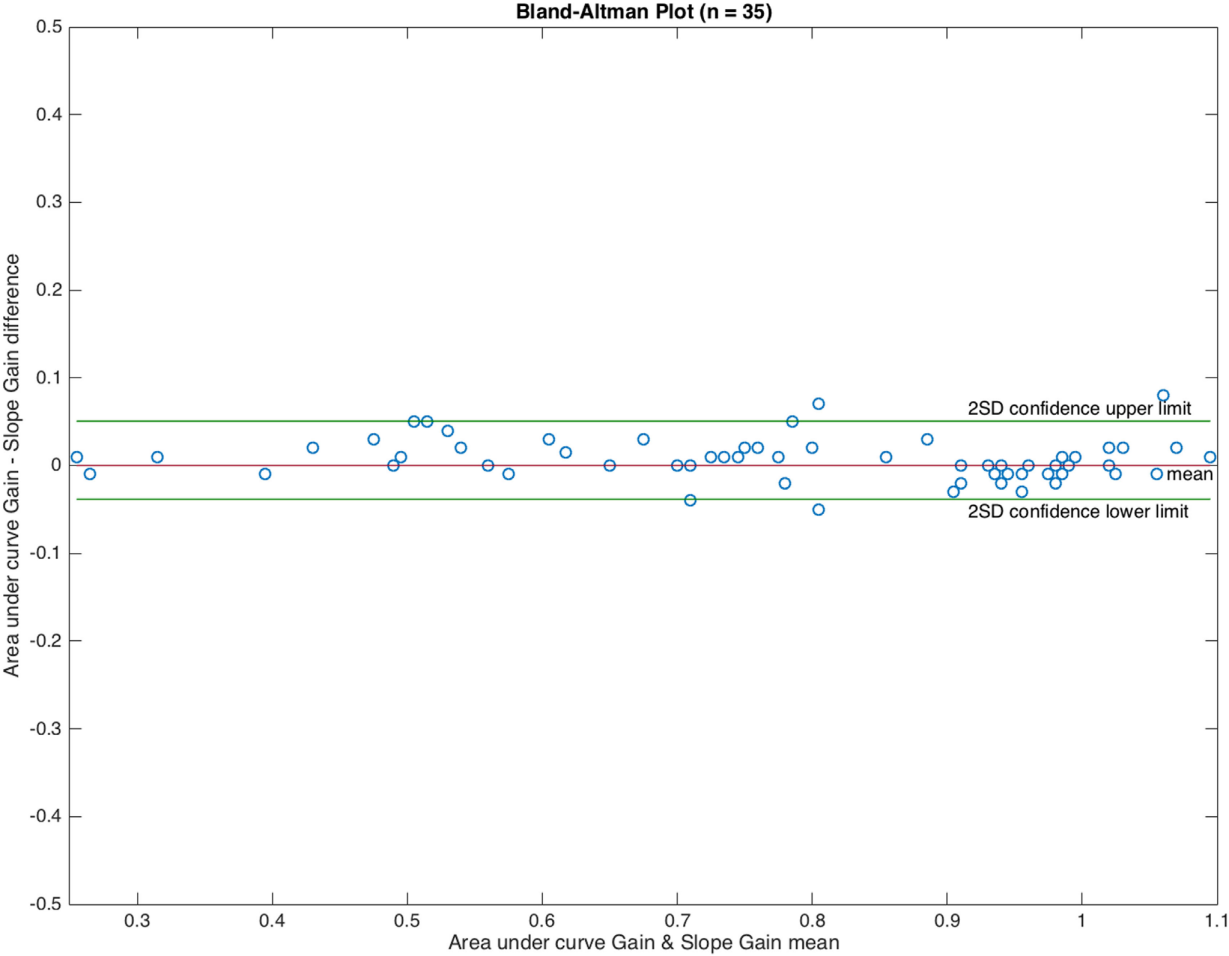
Bland and Altman plot showing graphical concordance between the two methods used to measure visually enhanced VOR gain. The differences observed between both methods were uniformly distributed in ±0.08 gain units. The intra-class correlation coefficient was 0.99.

The intra-class class correlation coefficient for positive G_AUC_ and G_SP_, and negative G_AUC_ and G_SP_ was 0.99, with an upper limit of 0.99 and a lower limit of 0.99 (*p* < 0.001). The Bland and Altman plot for the graphical concordance evaluation is shown in Figure 5.

From this point onward, we only use the results obtained from the AUC method.

Inter-group ANOVA test for VVOR gain value obtained a statistical power of 82.17% assuming balanced groups and 95.27% assuming unbalanced groups, statistical power was calculated with a grouping variable with 3° of freedom and a measured effect size of 0.61 on ANOVA test for this grouping variable.

#### Healthy Subjects

**Figure 6:**
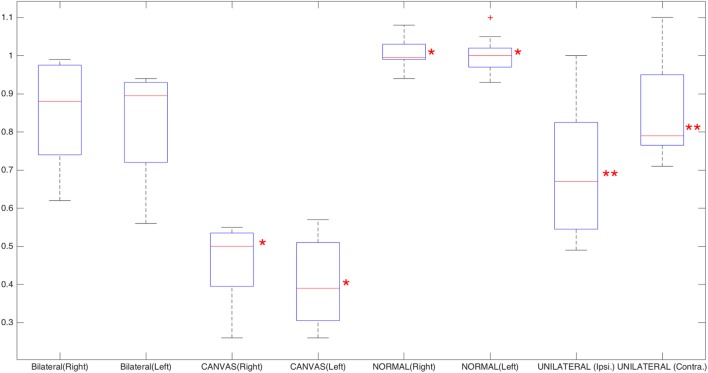
Box plot of the leftward and rightward visually enhanced VOR gains for normal, cerebellar ataxia, neuropathy, and vestibular areflexia syndrome (CANVAS), and bilateral vestibulopathy groups; for unilateral vestibulopathy group, operated (neurectomy) and non-operated sides have been plotted. Red asterisks mark the CANVAS and healthy groups; these two groups show significant statistical differences when compared to all the other groups, based on analysis of variance statistical method. Double red asterisks mark the differences between the operated and non-operated side in the unilateral vestibulopathy group (Wilcoxon test). The red crosses are outliers.

**Figure 7:**
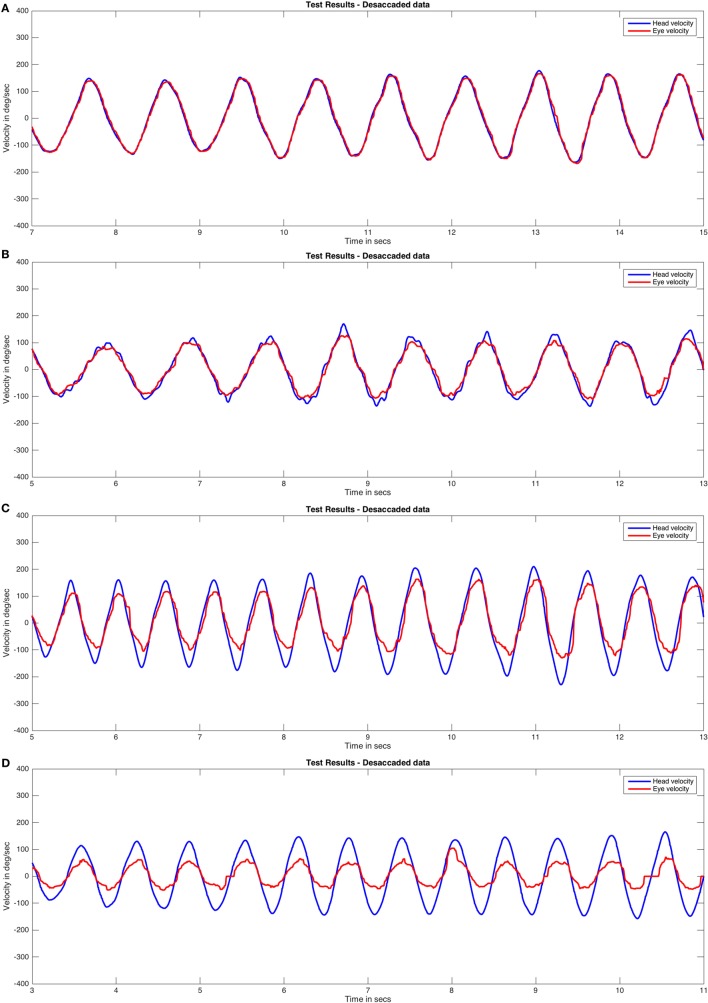
Visually enhanced VOR (VVOR) test responses by group (desaccaded eye velocity data). Cases closest to mean VVOR gain values were plotted: healthy group **(A)**, bilateral group **(B)**, unilateral group with a right vestibular hypofunction **(C)**, and cerebellar ataxia, neuropathy, and vestibular areflexia syndrome group **(D)**. Eye velocity plots were inverted to visually match the head velocity plots.

The mean positive (G_AUCp_) and negative (G_AUCn_) VVOR gains for healthy subjects were 1.00 ± 0.03 and 1.00 ± 0.05 (mean ± SD), respectively (Figure 6). A representative result is shown in Figure 7A.

##### Intra-Group Tests

No differences were obtained between the positive (G_AUCp_) and negative (G_AUCn_) VVOR gain values (*p* = 0.72). Significant statistical differences were obtained between the left and right values of VOR gain (*p* = 0.02).

##### Inter-Group Tests

Differences were statistically significant for both positive (G_AUCp_) and negative (G_AUCn_) VVOR gain compared to the positive (G_AUCp_) and negative (G_AUCn_) VVOR values of the other (pathologic) groups (*p* = 0.002 for positive VVOR gain and *p* < 0.001 for negative VVOR gain).

The ANOVA revealed a significant difference between the healthy group and all other groups for positive (G_AUCp_) (Table 3) and negative (G_AUCn_) (Table 4) VVOR gain values.

**Table 3:** Results obtained from one-factor analysis of variance statistical method (ANOVA) (with Bonferroni *post hoc* test) of positive visually enhanced VOR (VVOR) gain values.

Differences in positive VVOR gain (G_AUCp_) (ANOVA)				

Groups	Healthy	Vestibular bilateral	Vestibular unilateral	CANVAS
Healthy	–	0.16 ± 0.05 (*p = 0.01)	0.19 ± 0.1 (*p = 0.005)	0.55 ± 0.06 (*p < 0.001)
Bilateral vestibular	−0.16 ± 0.05 (*p = 0.01)	–	0.03 ± 0.05 (*p* = 1)	0.38 ± 0.06 (*p < 0.001)
Unilateral vestibular	−0.19 ± 0.05 (*p = 0.005)	−0.03 ± 0.05 (*p* = 1)	–	0.35 ± 0.06 (*p < 001)
CANVAS	−0.55 ± 0.06 (*p < 0.001)	−0.38 ± 0.06 (*p < 0.001)	−0.35 ± 0.06 (*p < 0.001)	–

*ANOVA revealed statistical significant difference between the positive VVOR gain variable for the groups used in this study (*p* < 0.001)*.

* represents significant difference (*p* < 0.05).

*CANVAS, cerebellar ataxia, neuropathy, and vestibular areflexia syndrome*.

**Table 4:** Results obtained from one-factor analysis of variance statistical method (ANOVA) (with Bonferroni *post hoc* test) for negative visually enhanced VOR (VVOR) gain values.

Differences in negative VVOR GAIN (G_AUCn_) (ANOVA)				

Groups	Healthy	Vestibular bilateral	Vestibular unilateral	CANVAS
Healthy	–	0.17 ± 0.05 (*p = 0.02)	0.26 ± 0.06 (*p = 0.001)	0.59 ± 0.07 (*p < 0.001)
Bilateral vestibular	−0.17 ± 0.05 (*p = 0.02)	–	0.08 ± 0.06 (*p* = 1)	0.41 ± 0.07 (*p < 0.001)
Unilateral vestibular	−0.26 ± 0.06 (*p = 0.001)	−0.08 ± 0.06 (*p* = 1)	–	0.33 ± 0.07 (*p < 0.001)
CANVAS	−0.59 ± 0.07 (*p < 0.001)	−0.41 ± 0.07 (*p < 0.001)	−0.33 ± 0.07 (*p < 0.001)	–

*ANOVA revealed statistical significant differences between the negative VVOR gain variable for the groups used in this study (*p* < 0.001)*.

* represents significant difference (*p* < 0.05).

*CANVAS, cerebellar ataxia, neuropathy, and vestibular areflexia syndrome*.

#### Peripheral Bilateral Vestibulopathy

The mean positive and negative VVOR gains for patients with peripheral bilateral vestibulopathy were 0.84 ± 0.13 and 0.82 ± 0.15, respectively (Figure 6). A r epresentative result is shown in Figure 7B.

##### Intra-Group Tests

No differences were found between the positive and negative VVOR gain values (*p* = 0.16). No differences were found between the left and right VOR gain values (*p* = 0.67).

##### Inter-Group Tests

Analysis of variance statistical method indicated that the positive (Table 3) and negative (Table 4) VVOR gains were significantly different from those of the healthy and CANVAS groups. No significant difference with the unilateral vestibulopathy group was found.

#### Unilateral Vestibular Hypofunction

The mean positive and negative VVOR gains for patients with unilateral vestibular hypofunction were 0.69 ± 0.17 and 0.85 ± 0.13, for operated (neurectomy) and non-operated sides, respectively (Figure 6). A representative result for a patient with right-sided vestibular neurectomy is shown in Figure 7C.

##### Intra-Group Tests

Operated and non-operated VVOR gains were significantly different (*p* = 0.012, Wilcoxon test), as were the left and right VOR gains (*p* = 0.019, Wilcoxon test).

**Figure 8:**
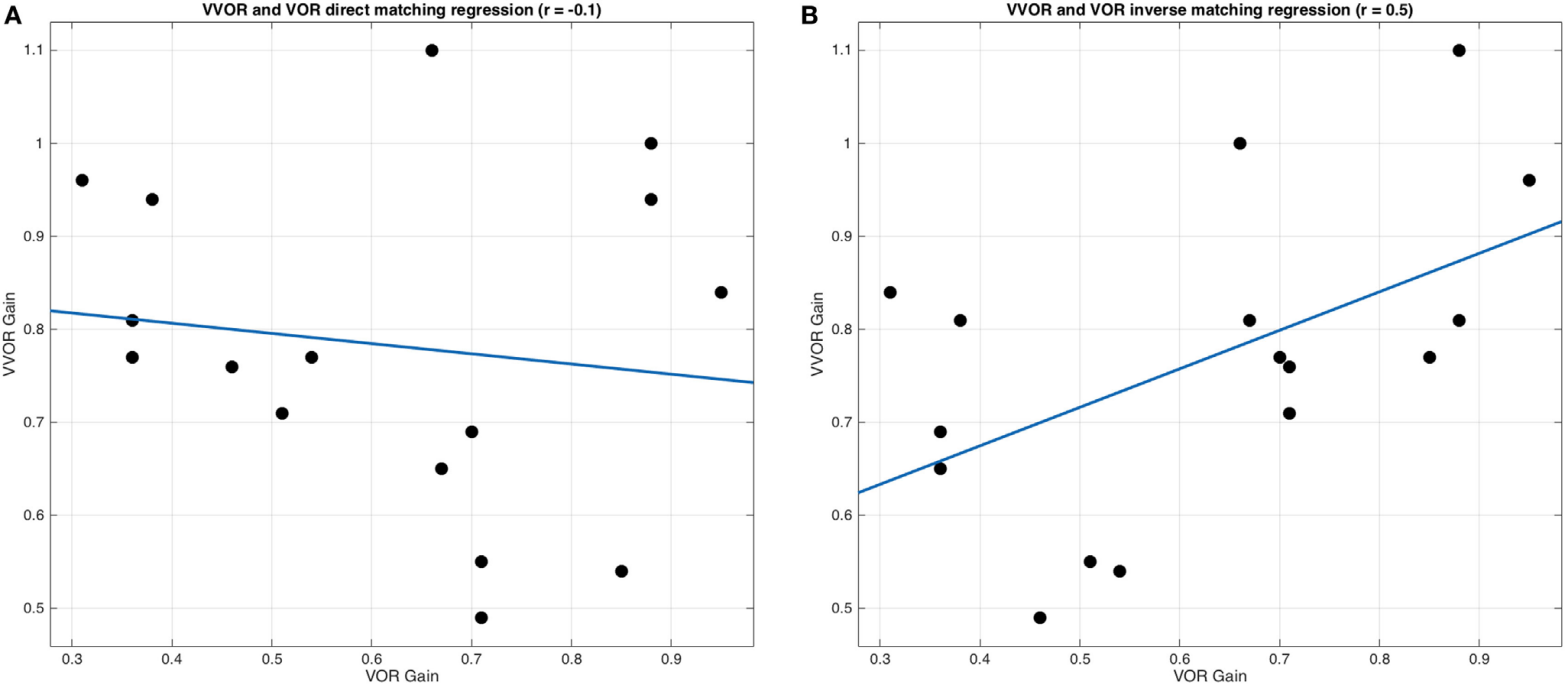
Linear regression analysis between visually enhanced VOR (VVOR) and vestibulo–ocular reflex (VOR) gain values. **(A)** Linear regression and scatter data are plotted, showing no significant and weak inverse correlation (Pearson’s *r* = −0.13, *p* = 0.63) between VVOR gain values and VOR gain values, assuming that positive values obtained with video head impulse test device while performing the VVOR test were representing rightward movements and negative values were representing leftward movements. **(B)** Linear regression and scatter data are plotted, showing a moderate and significant correlation (Pearson’s *r* = 0.52, *p* = 0.03) between VVOR gain values and VOR gain values, assuming that positive values were representing leftward head movements and negative values were representing rightward head movements. The blue line represents regression and black dots represent VVOR and VOR gain values.

For unilateral VVOR and VOR gains, a Pearson’s correlation of *r* = −0.13 (*p* = 0.63) was obtained for cases when VVOR direction and VOR direction in the vHIT matched (Figure 8A). However, when the inverse was considered, Pearson’s correlation was *r* = 0.52 (*p* = 0.03) (Figure 8B).

##### Inter-Group Tests

Analysis of variance statistical method indicated a significant difference between positive (Table 3) and negative (Table 4) VVOR gain values obtained from healthy subjects and patients with CANVAS.

#### CANVAS Group

The mean positive and negative VVOR gains for patients with CANVAS were 0.45 ± 0.11 and 0.4 ± 0.12, respectively (Figure 6). A representative result is shown in Figure 7D.

##### Intra-Group Tests

No differences were found between the positive and negative VVOR gain values (*p* = 0.27 on Wilcoxon test). No differences were found between the left and right VOR gain values (*p* = 0.22 on Wilcoxon test).

##### Inter-Group Tests

Significant differences were obtained for positive (Table 3) and negative VVOR gains (Table 4) of the CANVAS group, compared to those of the three other groups, i.e., healthy, peripheral bilateral, and unilateral peripheral vestibulopathy, using ANOVA.

#### Saccadic Analysis

The highest number of saccades per cycle was obtained for the patients with CANVAS. However, only a tendency toward a statistically significant difference was observed between the CANVAS group and other groups (*p* = 0.07) and with patients in the unilateral peripheral vestibulopathy group (*p* = 0.08).

#### Head Oscillation Frequency

We found that the frequency of oscillation was significantly lower (*p* = 0.04) in patients with peripheral bilateral vestibulopathy (mean = 1 ± 0.48 Hz) compared to that of healthy subjects and other patients (1.57 ± 0.37 Hz).

All the original results obtained in this study can be downloaded from www.mlibra.com/Descargas/RESULTS.zip and are also available as supplementary material for this manuscript.

## Discussion

The key factors for developing an automated methodology for VVOR gain measurement were analyzing head and eye movements as a virtually periodic signal and removing the fast phases of nystagmus elicited during testing. To accomplish this, we applied a median one-dimensional adaptive filter to the eye movement data. This filter analyzes the data in a stepwise manner, like a Turing machine. For each value, the analysis reads the nearest data in a pre-determined window and determines if the actual value is outside the median of this window. If the value is outside the median, it is corrected and the filter moves on to the next value (16). The two main issues to consider when using this type of filter are first, the optimal window size required to achieve appreciable data correction and second, avoidance of artifacts resulting from the modification of the data by the applied filter. After a detailed analysis of the graphical result were obtained for each patient (Figure 4), we found that only in the events of maximal head velocity was there any evidence of smoothing of the eye velocity data (i.e., the plot’s trace on local peaks is wider than that in other parts of the curves), both for the RAW and desaccaded data. This could be related to an artifact caused by errors in pupil tracking (25), which could influence the selection of the gain analysis method. The values obtained from the healthy group in our study support that this artifact is probably not significantly affecting our analysis method. The actual gain (1.00 ± 0.03 rightward and 1.00 ± 0.05 leftward) is not only close to the expected gain value of 1, but is similar to the values reported by previous studies using other methods of VVOR recording (1).

In this study, we developed two mathematical methods to obtain the gain from eye and head velocity data of the VVOR tests: the area under the curve and the slope of the scatter plot. The intra-class correlation of 0.99 provides evidence that both methods are not only valid but also similar when estimating VVOR gain results.

From these methods, we obtained two gain values; a positive value for rightward head movements and a negative value for leftward head movements from the same test. The possibility to extend this pair of gain values to obtain a unique value with the combined results of the VVOR test was not considered in this study. However, it could be of interest for future studies.

Two other methods to measure VVOR gain, the fast Fourier transform and assessment of the peak head and eye velocity ratio, were also evaluated, but disregarded in this study. The fast Fourier method was discarded because of its inability to correctly evaluate the positive and negative direction asymmetries. Fourier transforms were described by Joseph Fourier (26) as the transformation of a periodic signal into the summation of a finite number of symmetric sinusoidal functions. The differences observed between the positive and negative parts of the VVOR signal could, therefore, be hidden during this transformation. Although the assessment of VOR gain during vHIT using the peak eye to head ratio method shows good concordance with the results obtained with the AUC method (27), we decided not to use the former to avoid artifacts given the results obtained from the analysis of the curves (28).

The asymmetry of head impulses obtained during the VVOR test could be a limitation of this study that was not fully controlled. The inclusion of feedback mechanisms to assist the examiner during the VVOR test, applied to ensure a better control of timing and amplitude parameters of the VVOR impulses, should be considered or evaluated for future investigations and the design of further medical devices.

In the present study, we obtained a significant difference in the VVOR gain values for both directions (rightward and leftward) between the healthy and CANVAS groups (Table 5). The healthy subjects had a gain close to 1, with no individuals having a gain less than 0.95. However, the VVOR gain for both directions in the CANVAS group was close to 0.5 or lower in all cases. It is important to mention that subjects in the healthy group were significantly younger, and that with increasing age, a small but significant decline in VVOR gain is observed (29). In the CANVAS group, VVOR gain was significantly lower than that for any other patient group, which positively correlates with clinical observations (6) and previous findings regarding the assessment of visual and vestibular interactions (1). In general, it represents the loss of the physiological synergistic nature of VOR and SP for the stabilization of the visual scene.

**Table 5:** Summary of vestibulo–ocular reflex (VOR) and visually enhanced VOR (VVOR) values obtained in this study for each group.

Parameters	Healthy	CANVAS	Vestibular bilateral	Vestibular unilateral
Mean gain VOR	Normal (bilateral) 1 ± 0	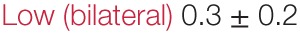	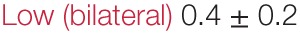	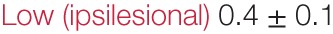
Mean gain VVOR	Normal (bilateral) 1 ± 0	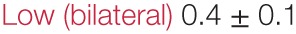	Mild (bilateral) 0.8 ± 0.1	Moderate (ipsilesional) 0.6 ± 0.1
Saccades per cycle VVOR	0 ± 0	4 ± 2	3 ± 1.6	2 ± 0.8

*Mean gain values are presented for VOR, and values of mean gain and saccades per cycle are presented for VVOR. CANVAS, cerebellar ataxia, neuropathy, and vestibular areflexia syndrome*.

*Red color is used to highlight lowest values on VOR and VVOR gain values*.

In the unilateral and bilateral vestibulopathy groups, the reduction in VVOR gain (for both directions) was mild or moderate (Table 5). Although VVOR gain was lower for the former group than for the latter, there were no significant differences between the two groups as would be expected when the VVI is functioning normally (30). The highest values obtained for the bilateral vestibulopathy group could be the consequence of five different phenomena. First, the marked heterogeneity in clinical diagnoses for the bilateral vestibulopathy group could contribute to this observation. It was recently shown that bilateral vestibular deficits are not uniform across different clinical diagnoses when the high-frequency and high-velocity tests are performed and the six semicircular canals are assessed (31). Second, these high values could be due to differences in the frequency of stimulation (1), as that used for patients with bilateral vestibulopathy was lower (~1 Hz) compared to that used for the unilateral vestibulopathy group (~1.5 Hz). Third, in the unilateral vestibulopathy group, disease etiology and treatment were homogeneous. To account for this, a specific and unique follow-up was performed and is mentioned below. Fourth, predictability has been reported as a cause of enhanced VOR responses observed in the autorotation test (32) and other eye–head movements (33). Despite VVOR being a passive test, predictability could contribute to the observed responses in the bilateral vestibular group. Finally, the fact that patients with peripheral bilateral vestibulopathy exhibit an adaptive improvement of SP could be one of the factors responsible for vestibular compensation (34).

In the unilateral vestibulopathy group, we found statistically significant differences between operated and non-operated sides for VVOR gain values. We also found that the VOR gain during the vHIT for leftward head impulses correlates with a positive (rightward head movement) VVOR gain. Similarly, there was a correlation of the vHIT results for rightward head impulses with negative (leftward head movements) VVOR gain. This correlation implies that VOR gain in the vHIT for head impulses toward the operated side correlate with VVOR gain when the head is moving toward the non-operated side. The cause for this mismatch is that the vHIT device used in this study records rightward head movements as a negative velocity and the leftward movements as a positive velocity such that VVOR gain values are positively correlated with the VOR gain values in the unilateral vestibulopathy group. This indicates the need for the industry to adopt standards that allow proper identification of the laterality of the records obtained by this class of devices. This mismatch was discovered “*a posteriori*” due to the results for the correlation and regression analysis of the data obtained from the unilateral vestibulopathy group. When we presented these data in Section “Results,” we considered to label it as true operated and non-operated sides, to avoid reader’s confusion in data interpretation.

In the design of this study, we did not consider including other groups of patients. Our study was limited to a reduced group of patients and did not account for other pathologies such as progressive supranuclear paralysis in which SP and OKN reflex are altered and normal VOR function is normal. Despite this shortcoming, this is the first study to collect results with the main objective of testing different analysis methods in the patient population. To include a group of patients with progressive supranuclear paralysis and other pathologies or a wider sample of normal subjects should be considered in further studies to enhance the results obtained in the present study.

Spontaneous nystagmus effects on the VVOR calculation method was not measured or evaluated in this study. In future studies, we recommend to not use the developed VVOR analysis method on patients who do not present visually suppressed spontaneous nystagmus.

Another factor to be considered in VVOR responses is the cervico–ocular reflex (COR). It is known that in healthy subjects, the contribution of COR to gaze stability is low (35). A similar finding could be expected in patients with CANVAS, as demonstrated by published reports of patients with bilateral vestibular loss, and altered SP and optokinetic function (similar to the CANVAS group) in which COR was not found to contribute significantly to gaze stability (7). However, in the vestibular bilateral group, it is possible that the COR serves a role in gaze stability for low frequency sinusoidal head movements, as has been previously reported (36). This could be another factor to consider during interpretation of the relatively high VVOR gain values obtained from this group.

In this study, the examiners were instructed to perform head movements in a 40–50° arch, with the maximal head velocity of 150–200°/s being reached at the neutral head–trunk position. This high head velocity could produce a VVOR response caused mainly by VOR, with a limited contribution of the OKN and SP reflexes (37). This could influence the results obtained in this study by producing a selective VOR response in the performed VVOR tests. Despite these inadequate pre-established velocity values, the maximal head velocities reached and measured in this study were significant: mean maximum head velocity value was 109.08 ± 33.89°/s and the mean frequency of rotation was 1.37 ± 0.47 Hz. These values of velocity are closer to the maximum head velocity reported in similar studies where combined OKN, SP, and VOR reflexes responses were ensured (1). For eliciting adequate VVOR responses with contributions of all reflexes, the recommended parameters are mean frequency of rotation of ~1 Hz and a maximal velocity of head movement of ~100°/s (1, 37).

In conclusion, we detailed two automated mathematical methods to measure the gain of VVOR that can be applied to the results of actual clinical tests conducted using relevant instruments. The VVOR gain values measured using the methods developed in this study are in concordance with recently published experimental results and appear to objectively characterize the VVOR alteration observed in patients with CANVAS. Furthermore, they are capable of distinguishing healthy subjects from patients with vestibular disorders.

## Ethics Statement

Written informed consent was obtained from all the patients. Since no novel or exceptional interventions were performed in this observational clinical study, only the approval of the local ethical committee for the corresponding institutions was required for the researchers. The study was designed and performed in accordance with the ethical guidelines of the 1975 Declaration of Helsinki.

## Author Contributions

JR-M and NP-F has designed and conducted this study and its theoretical methods, JR-M, NP-F, AB-C, EM, GT-R, and XA, have participated on data collection, analysis, and manuscript redaction. JR-M has developed the methods to analyze the VVOR.

## Conflict of Interest Statement

The authors declare that the research was conducted in the absence of any commercial or financial relationships that could be construed as a potential conflict of interest.

## Abbreviations

ANOVA, analysis of variance statistical method; AUC, area under curve VVOR analysis method; CANVAS, cerebellar ataxia, neuropathy, and vestibular areflexia syndrome; COR, cervico–ocular reflex; CSV, comma separated values file format; FFT, fast Fourier transform mathematical method; HORT, head-only rotational test; OKN, optokinetic reflex; RAW, original (unprocessed) data; SCP, scatter plot regression VVOR analysis method; SP, smooth pursuit; vHIT, video head impulse test; VOR, vestibulo–ocular reflex; VVI, visual and vestibular interaction; VVOR, visually enhanced VOR.
