# Supplementary figures and images for: Mathematical Methods for Measuring the Visually Enhanced Vestibulo–Ocular Reflex and Preliminary Results from Healthy Subjects and Patient Groups
Source: Front Neurol. 2018 Feb 12;9:69. doi: 10.3389/fneur.2018.00069 (PMC5816338; doi:10.3389/fneur.2018.00069)

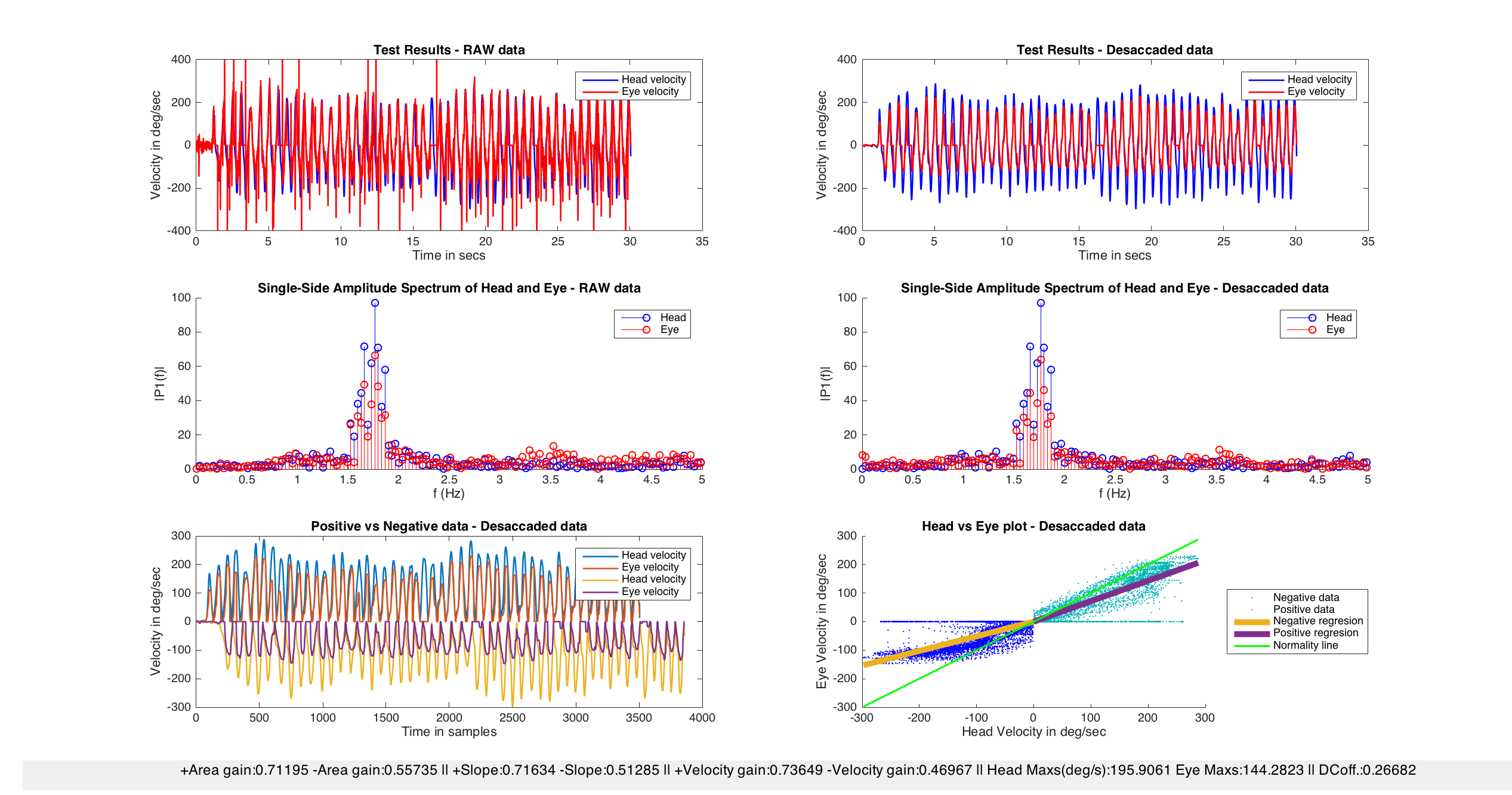

Supplement: Supplementary file 1 [file data_sheet_1.ZIP › RESULTS/PARTICIPANTS_TEST/A1.png]

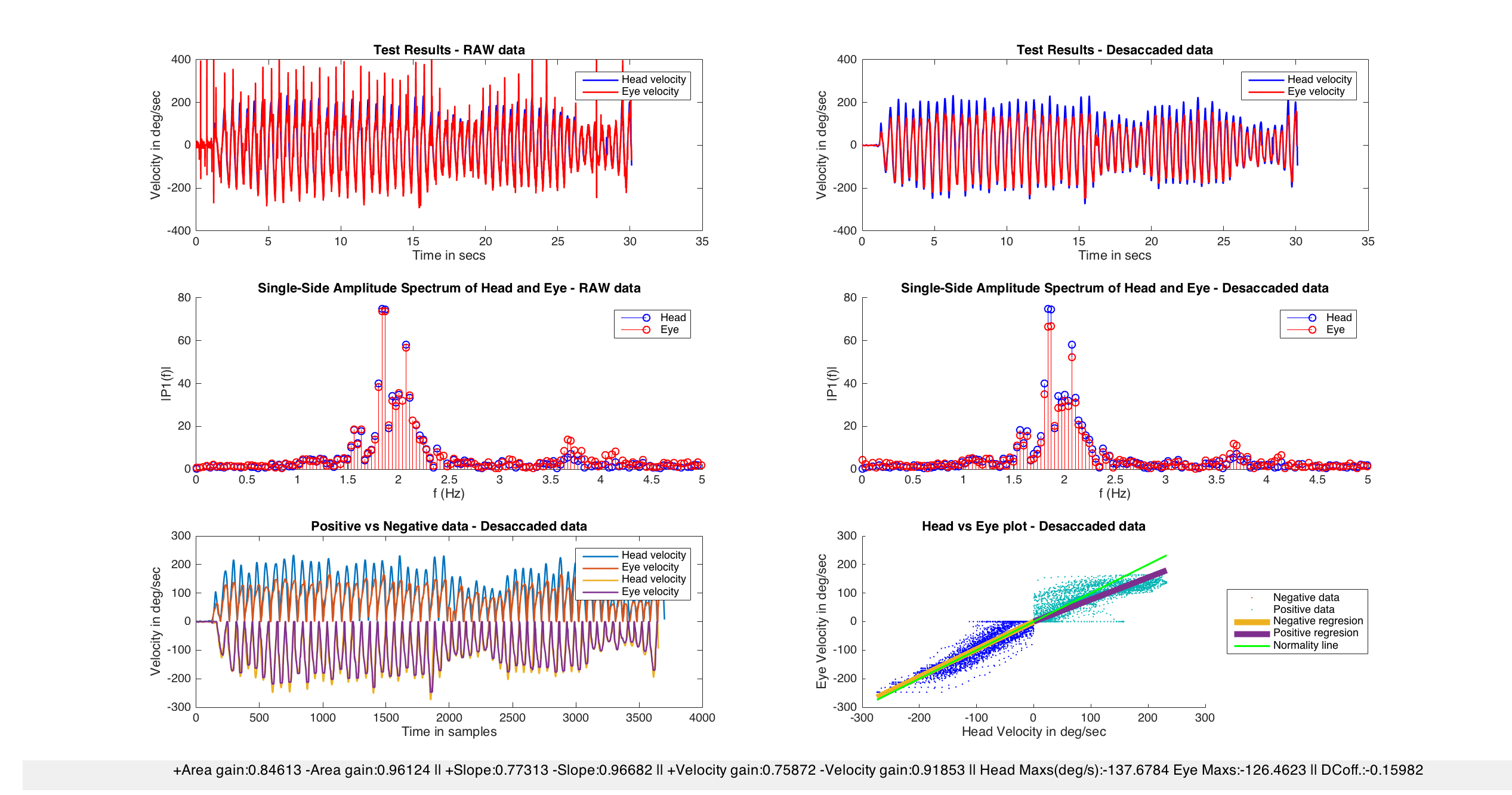

Supplement: Supplementary file 1 [file data_sheet_1.ZIP › RESULTS/PARTICIPANTS_TEST/A2.png]

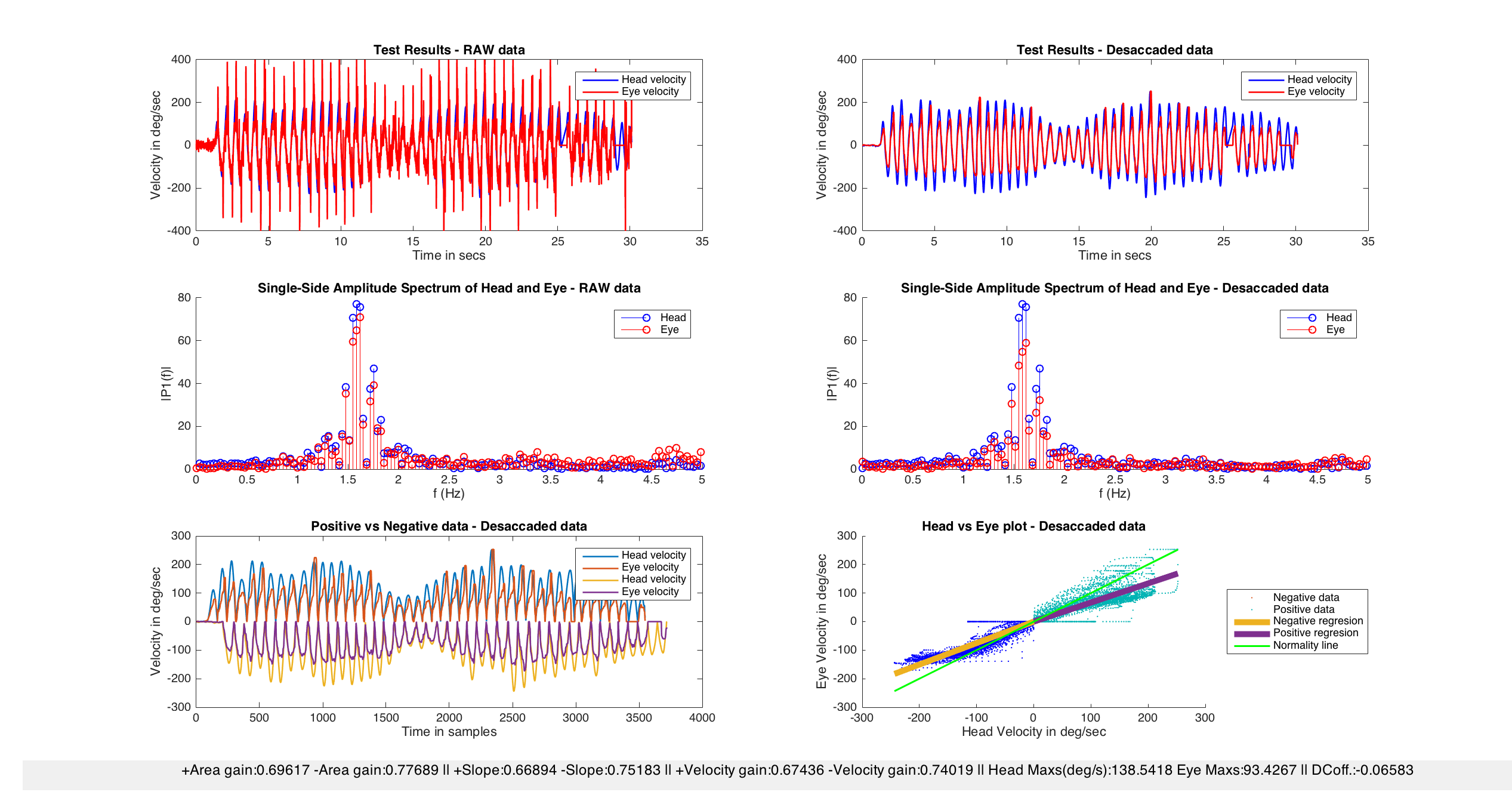

Supplement: Supplementary file 1 [file data_sheet_1.ZIP › RESULTS/PARTICIPANTS_TEST/A3.png]

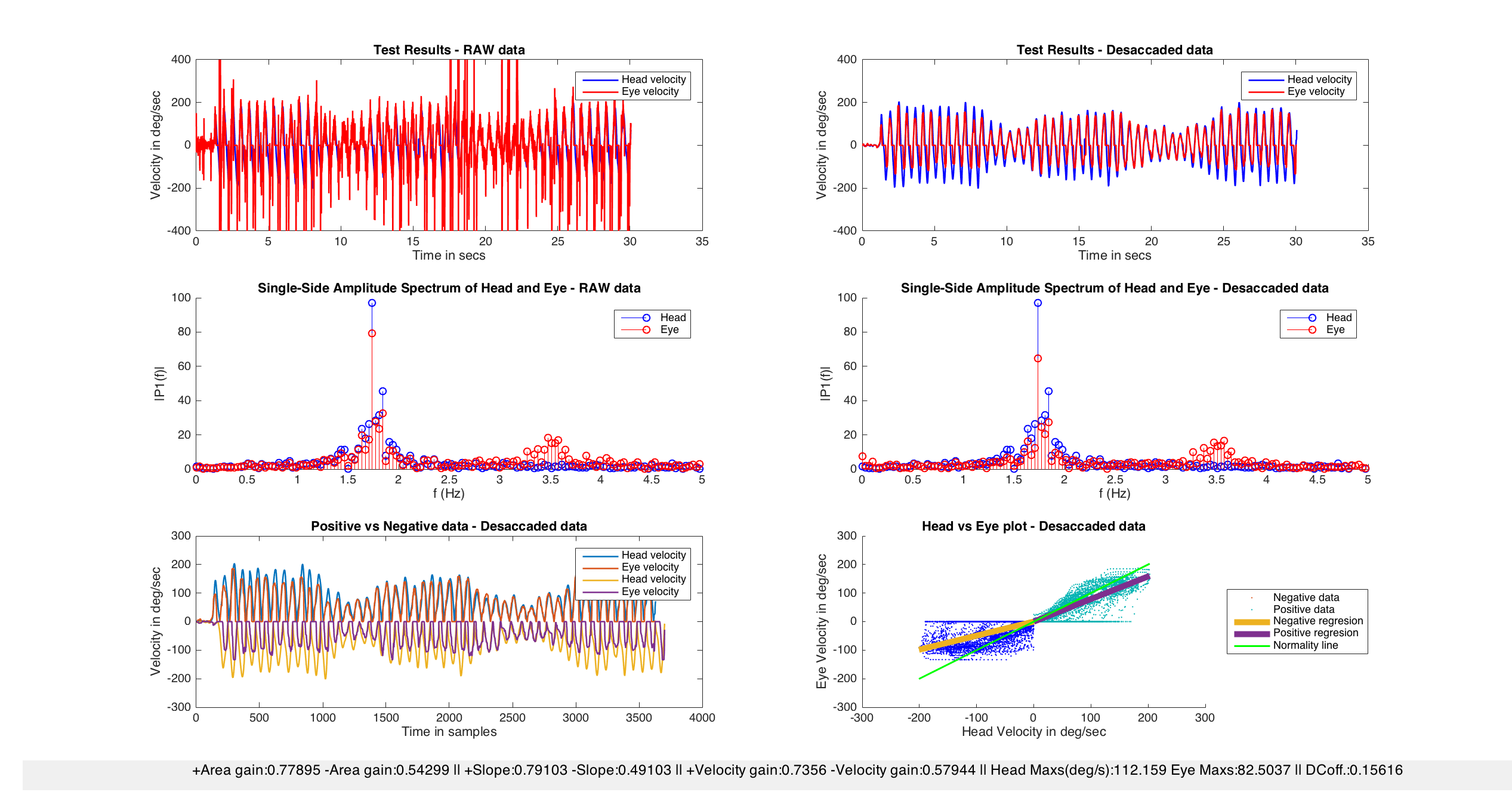

Supplement: Supplementary file 1 [file data_sheet_1.ZIP › RESULTS/PARTICIPANTS_TEST/A4.png]

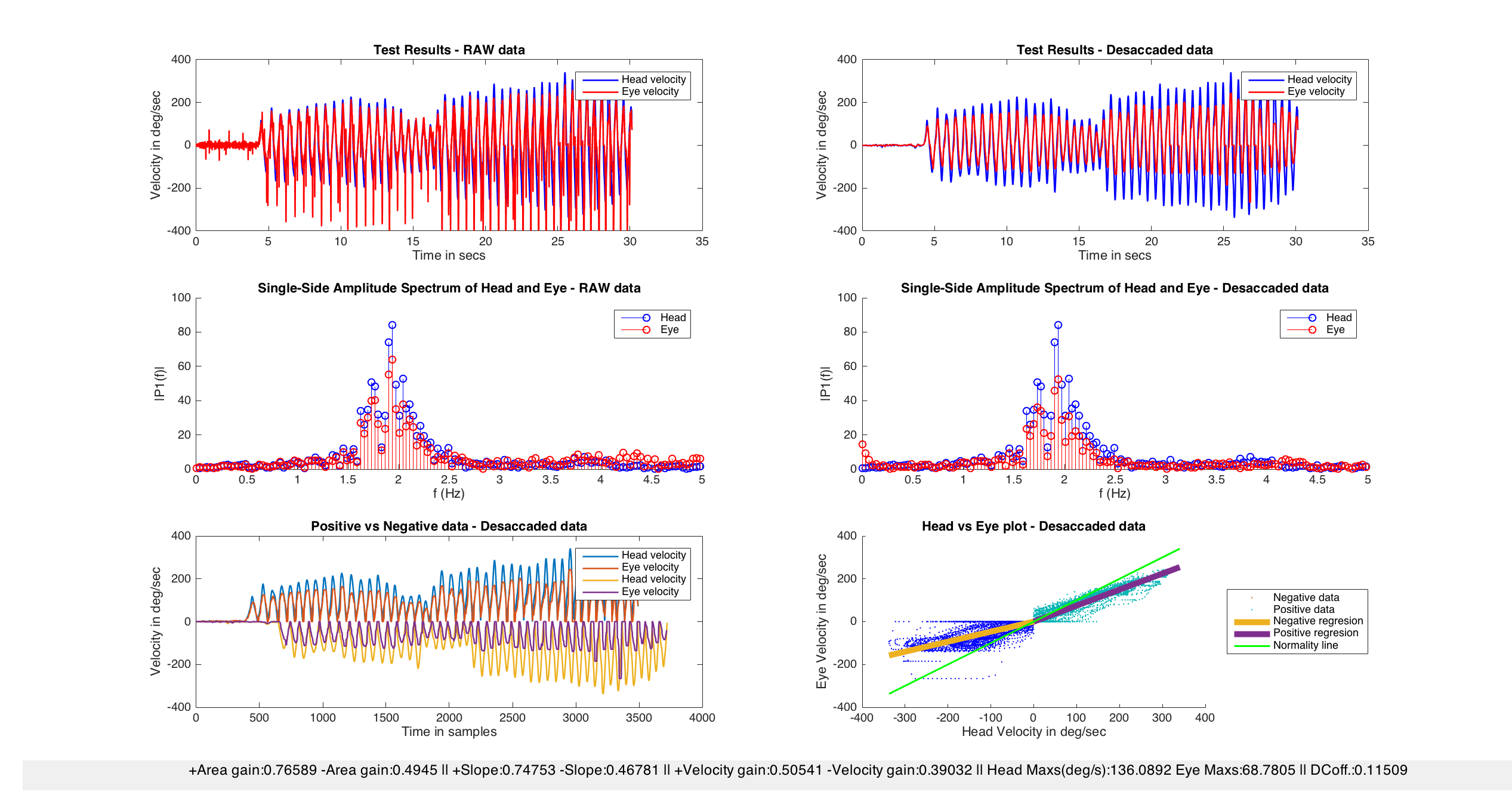

Supplement: Supplementary file 1 [file data_sheet_1.ZIP › RESULTS/PARTICIPANTS_TEST/A5.png]

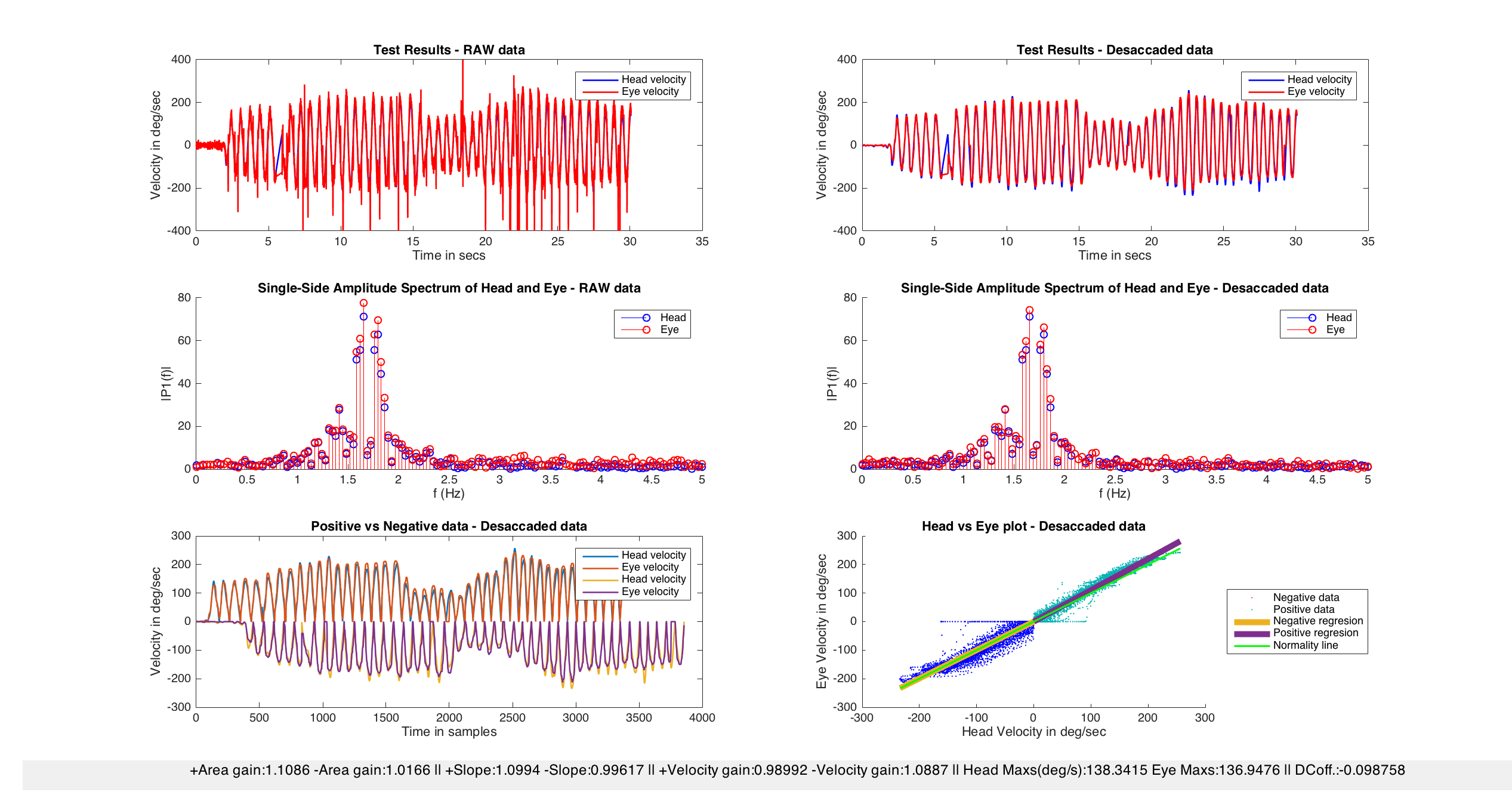

Supplement: Supplementary file 1 [file data_sheet_1.ZIP › RESULTS/PARTICIPANTS_TEST/A6.png]

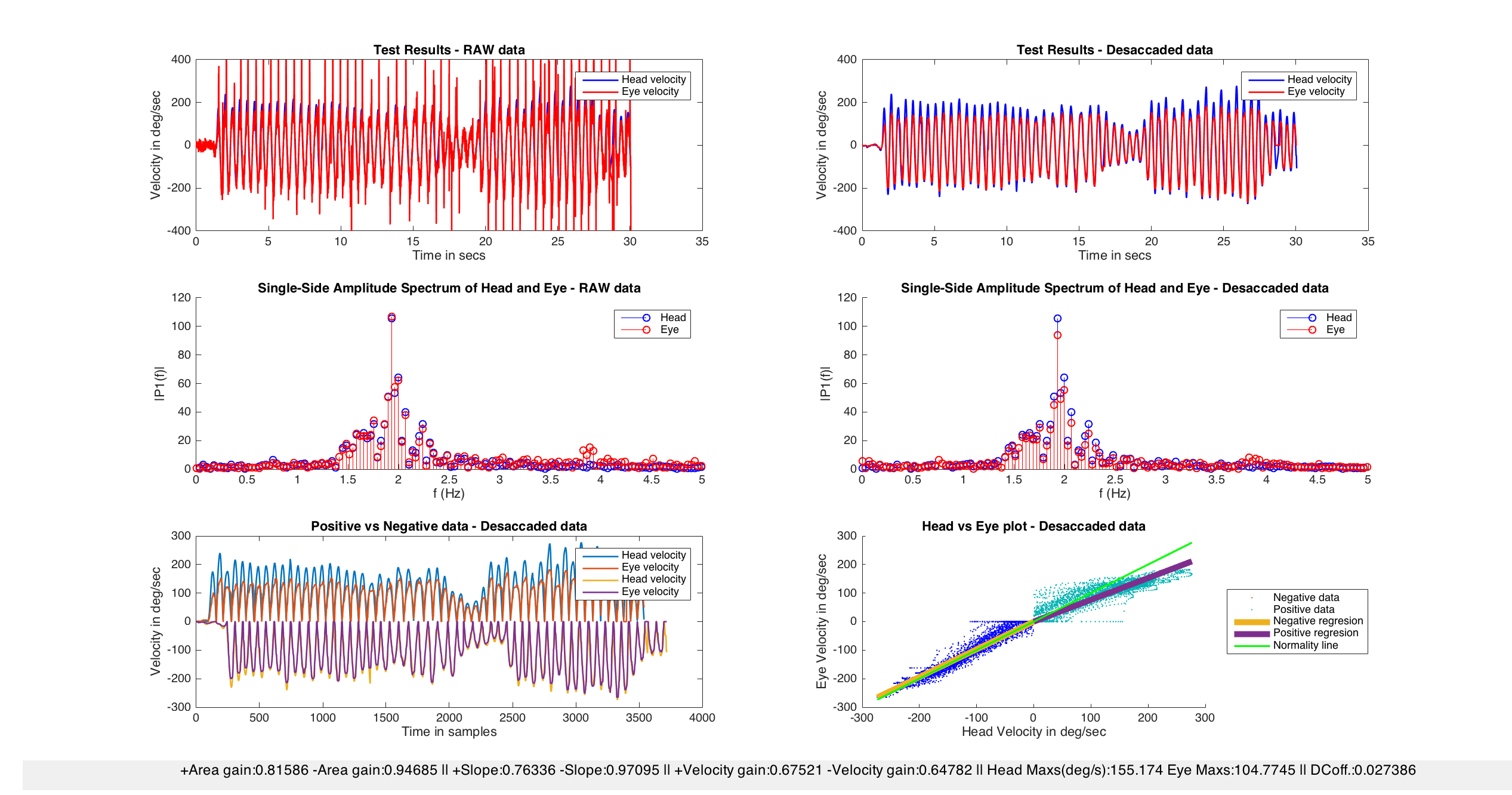

Supplement: Supplementary file 1 [file data_sheet_1.ZIP › RESULTS/PARTICIPANTS_TEST/A7.png]

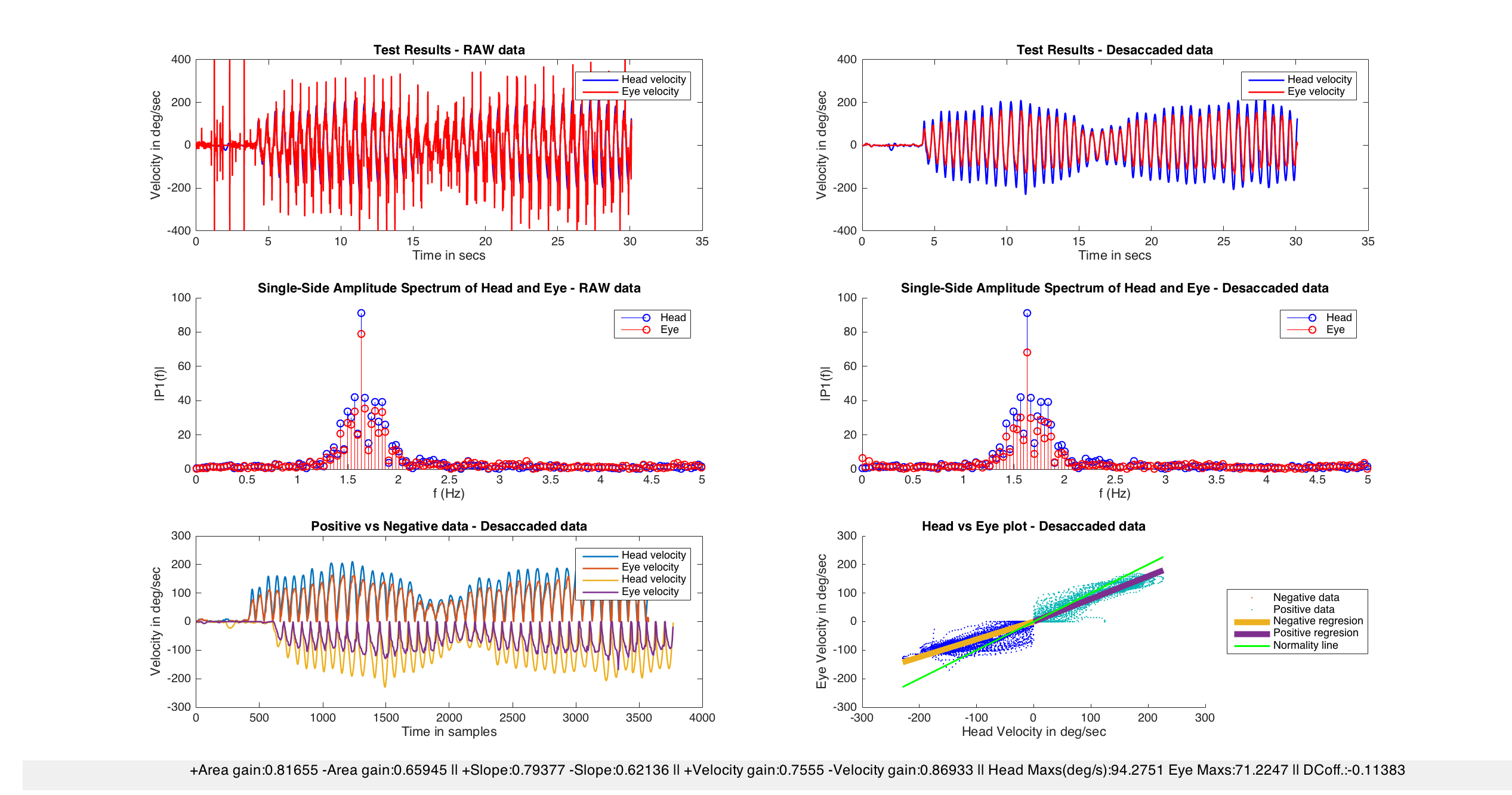

Supplement: Supplementary file 1 [file data_sheet_1.ZIP › RESULTS/PARTICIPANTS_TEST/A8.png]

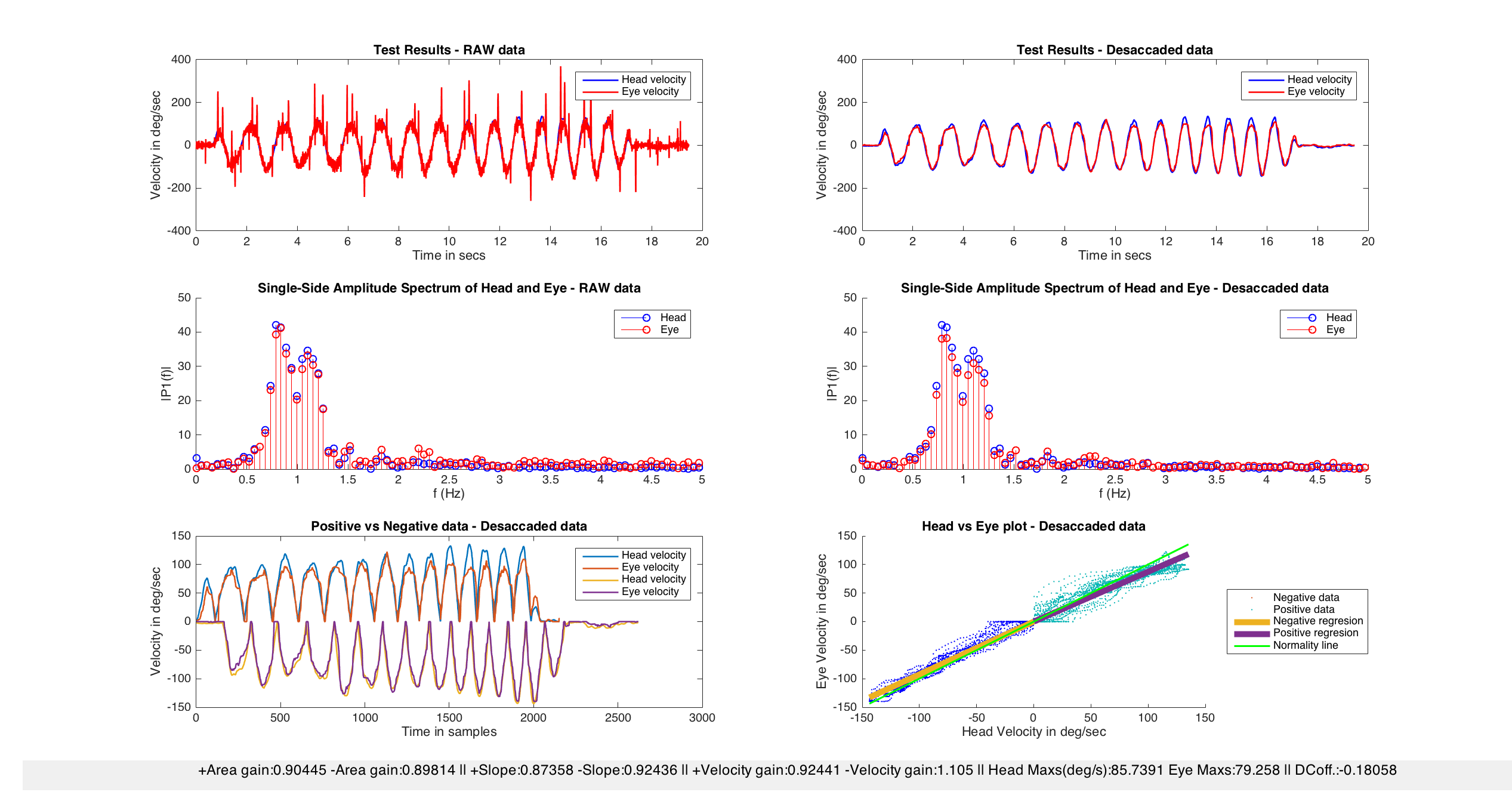

Supplement: Supplementary file 1 [file data_sheet_1.ZIP › RESULTS/PARTICIPANTS_TEST/B1.png]

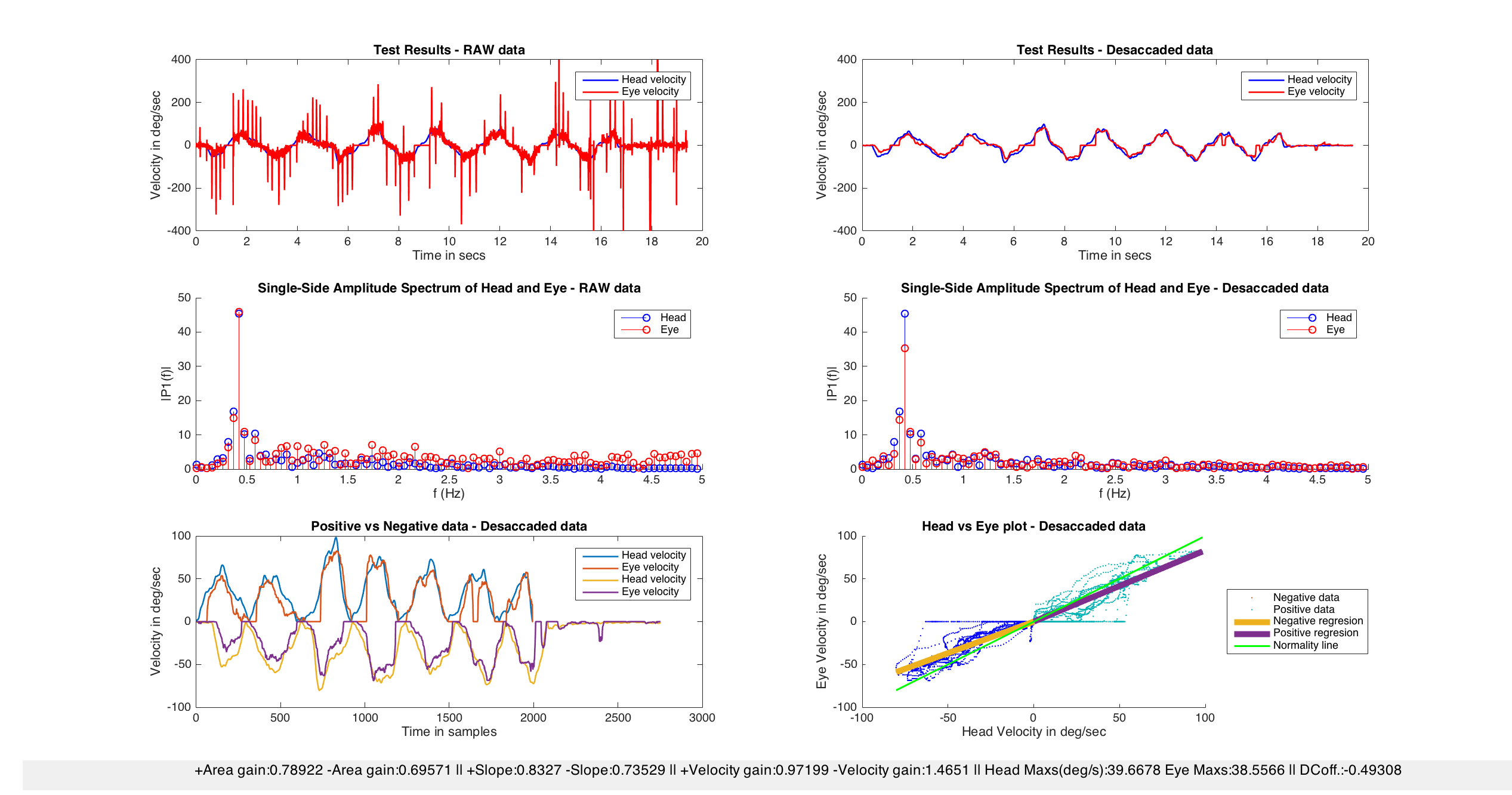

Supplement: Supplementary file 1 [file data_sheet_1.ZIP › RESULTS/PARTICIPANTS_TEST/B10.png]

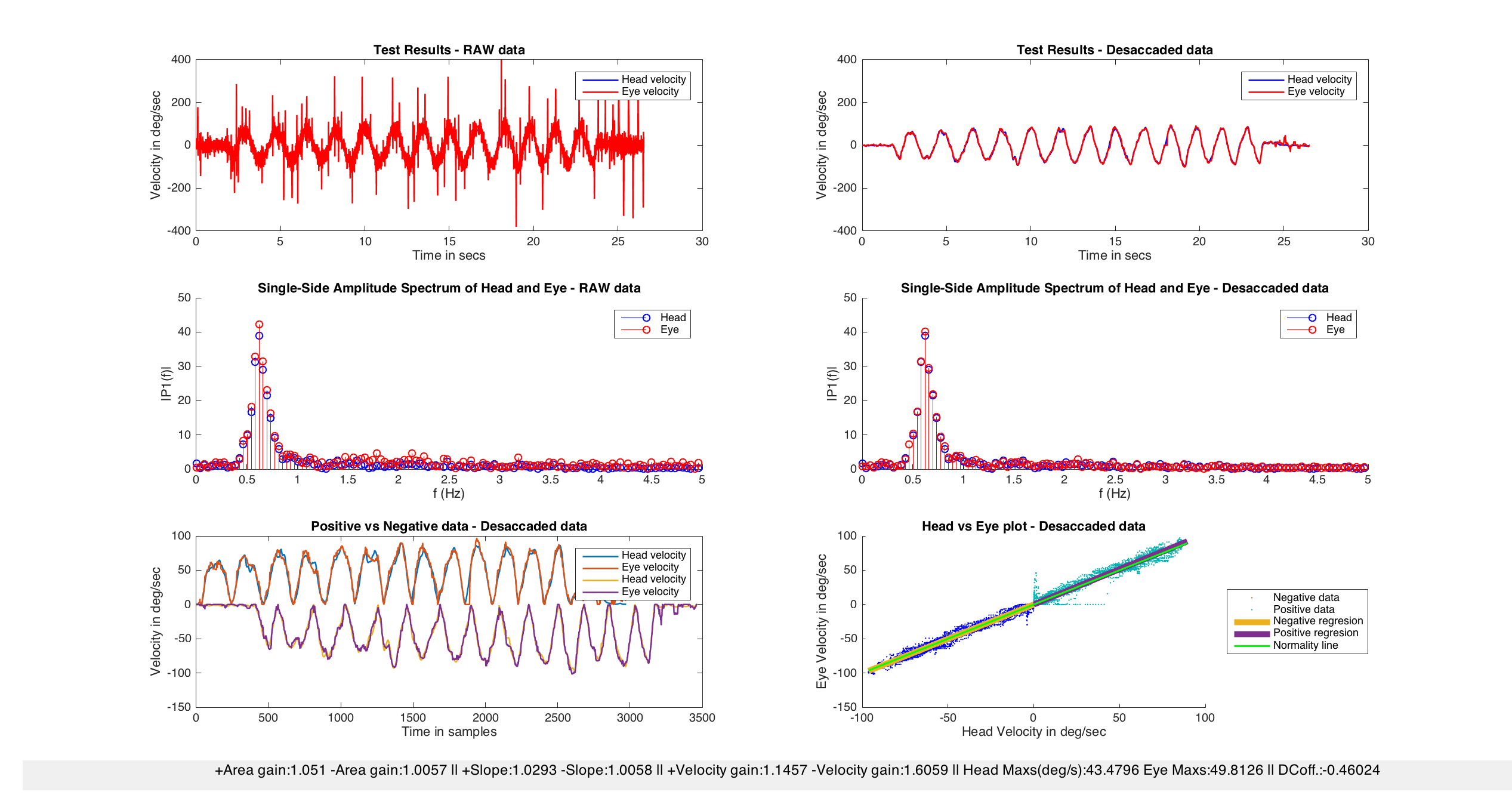

Supplement: Supplementary file 1 [file data_sheet_1.ZIP › RESULTS/PARTICIPANTS_TEST/B11.png]

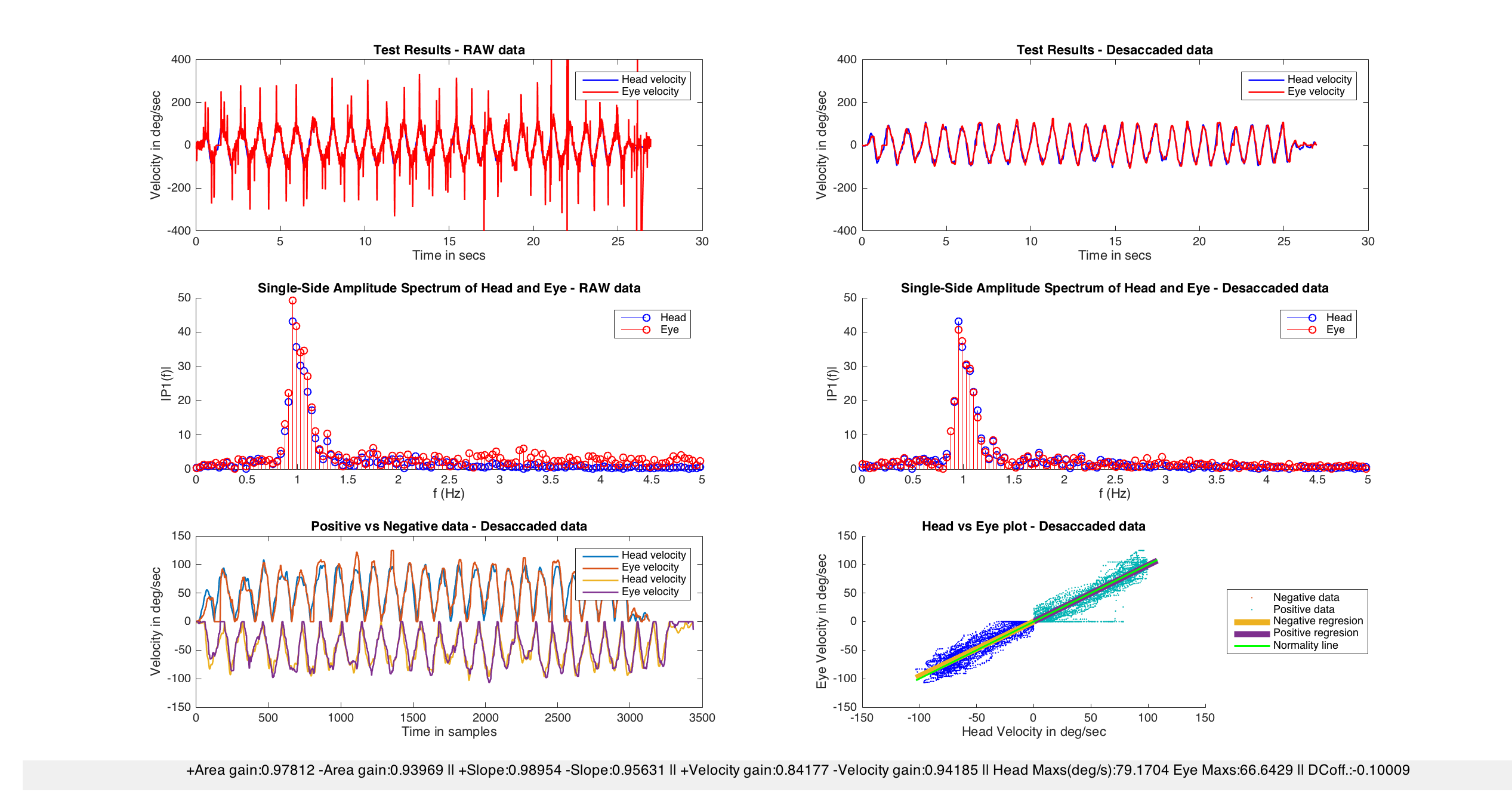

Supplement: Supplementary file 1 [file data_sheet_1.ZIP › RESULTS/PARTICIPANTS_TEST/B12.png]

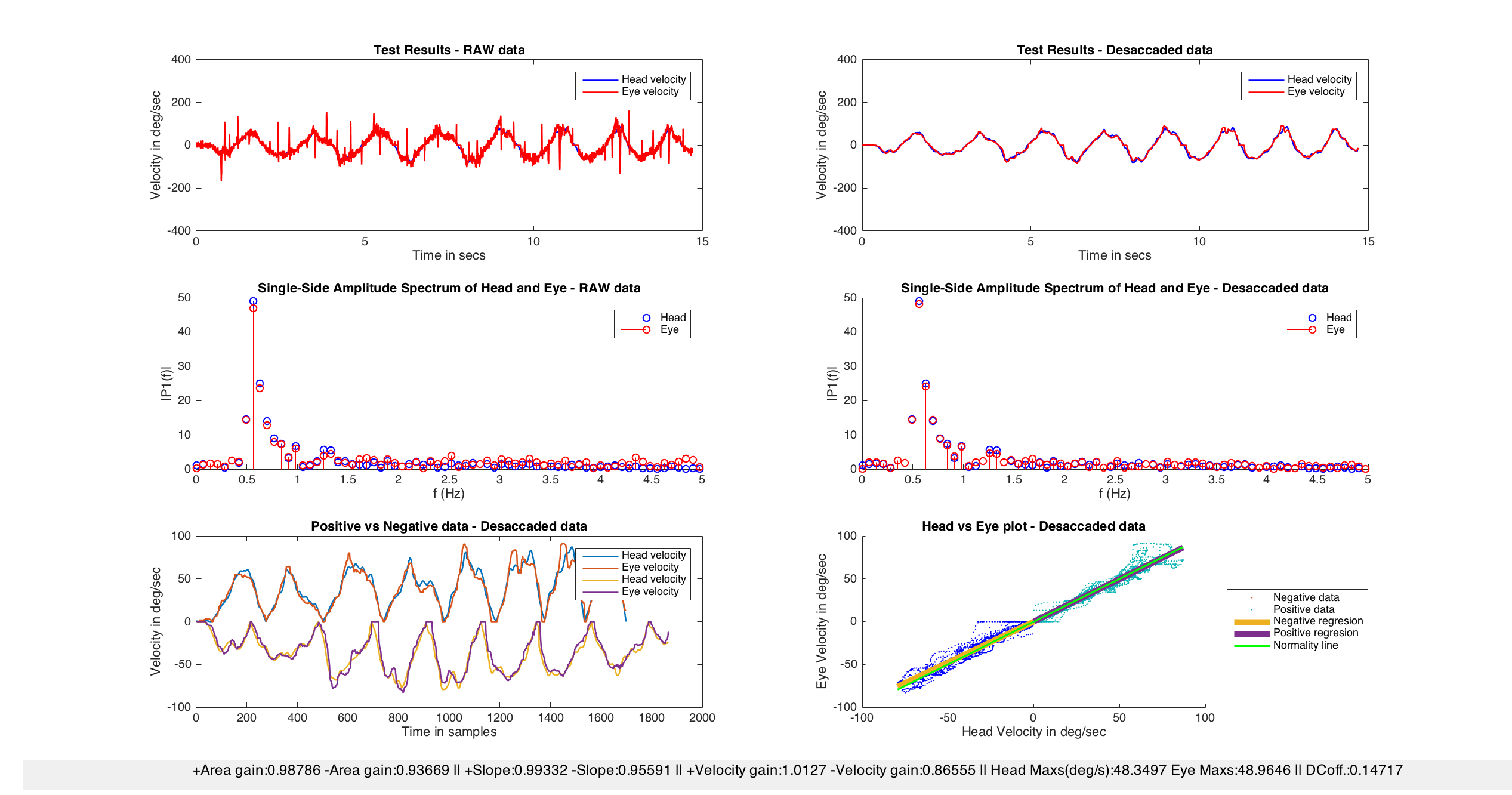

Supplement: Supplementary file 1 [file data_sheet_1.ZIP › RESULTS/PARTICIPANTS_TEST/B2.png]

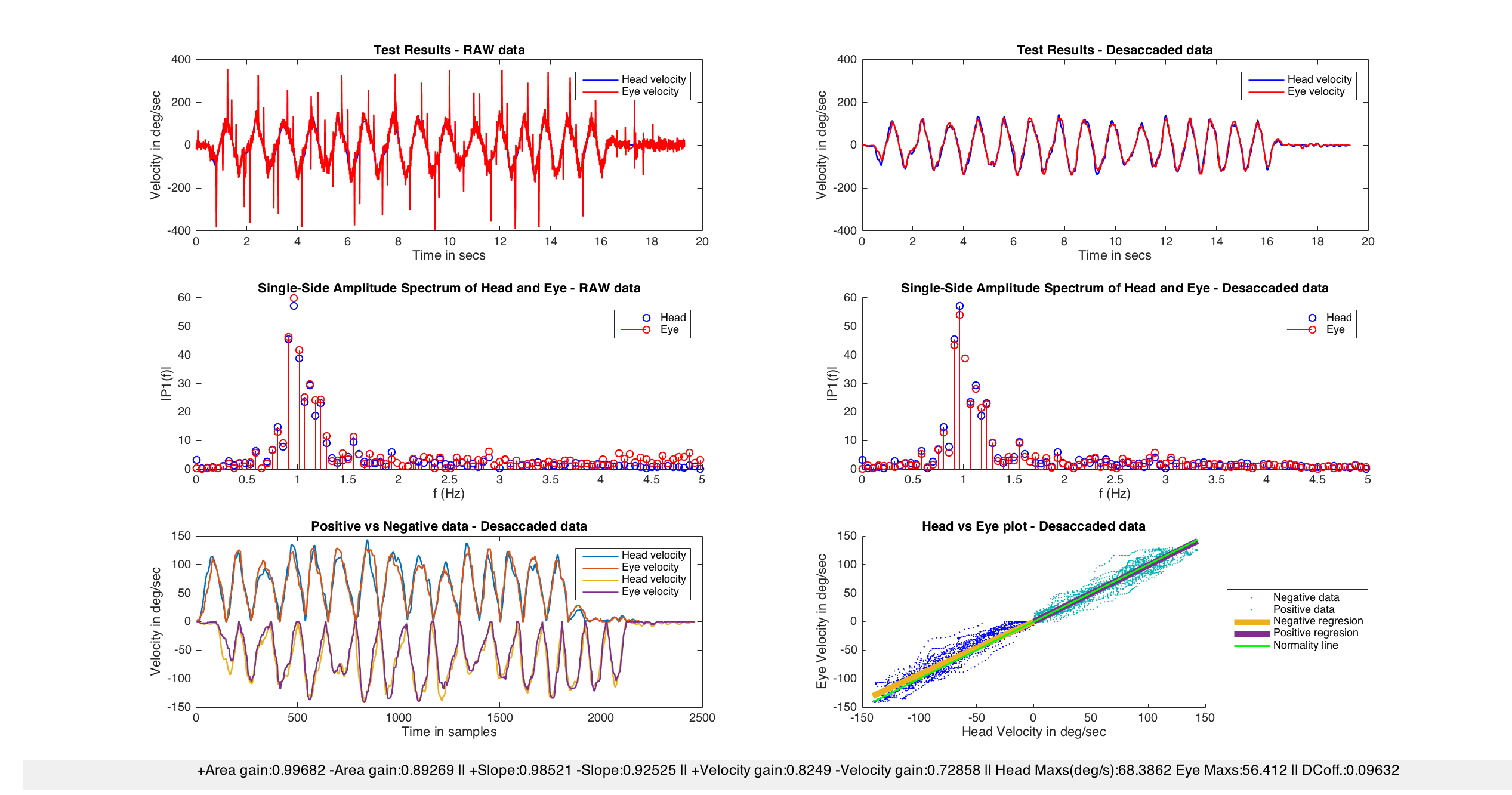

Supplement: Supplementary file 1 [file data_sheet_1.ZIP › RESULTS/PARTICIPANTS_TEST/B3.png]

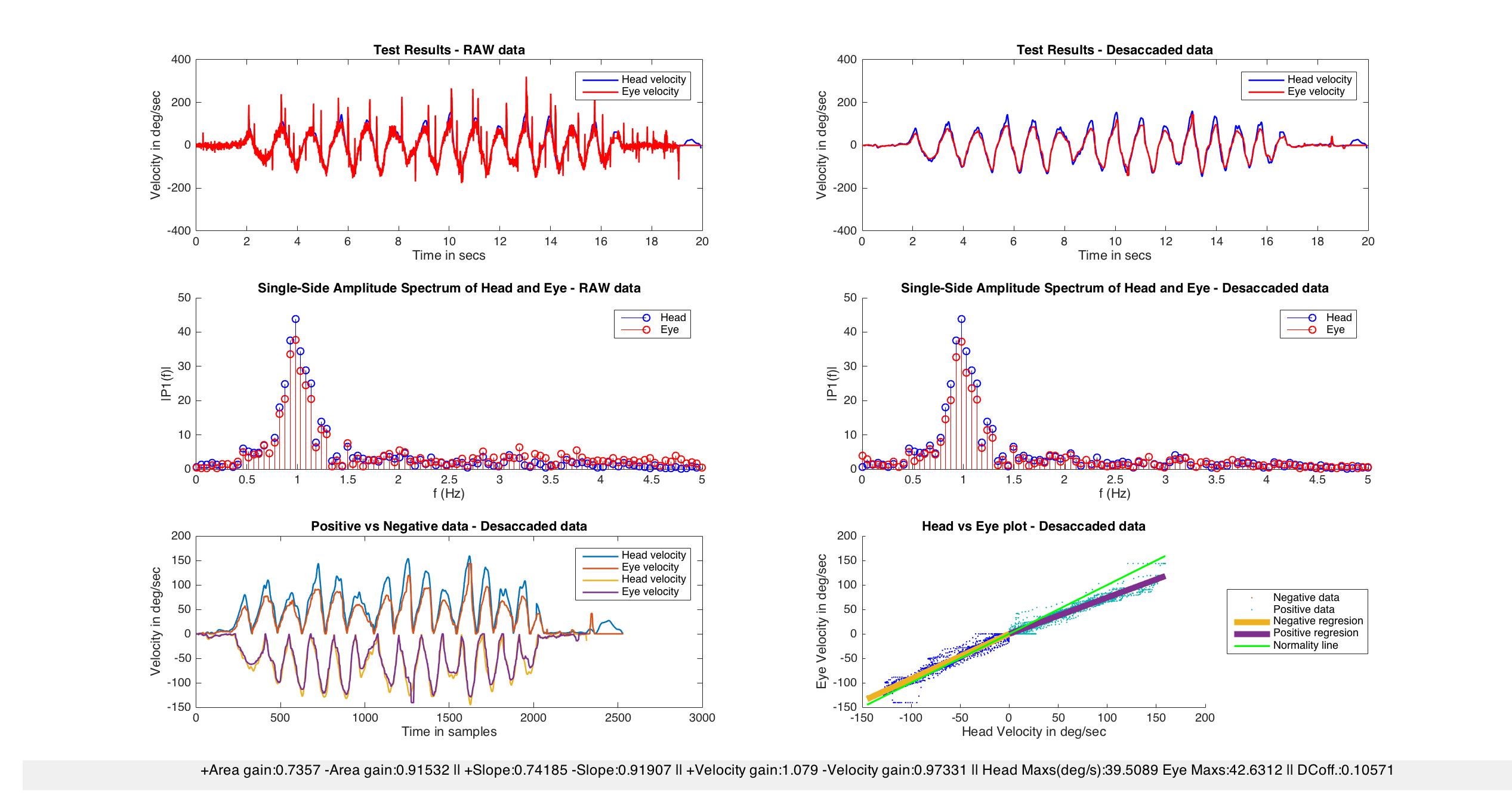

Supplement: Supplementary file 1 [file data_sheet_1.ZIP › RESULTS/PARTICIPANTS_TEST/B4.png]

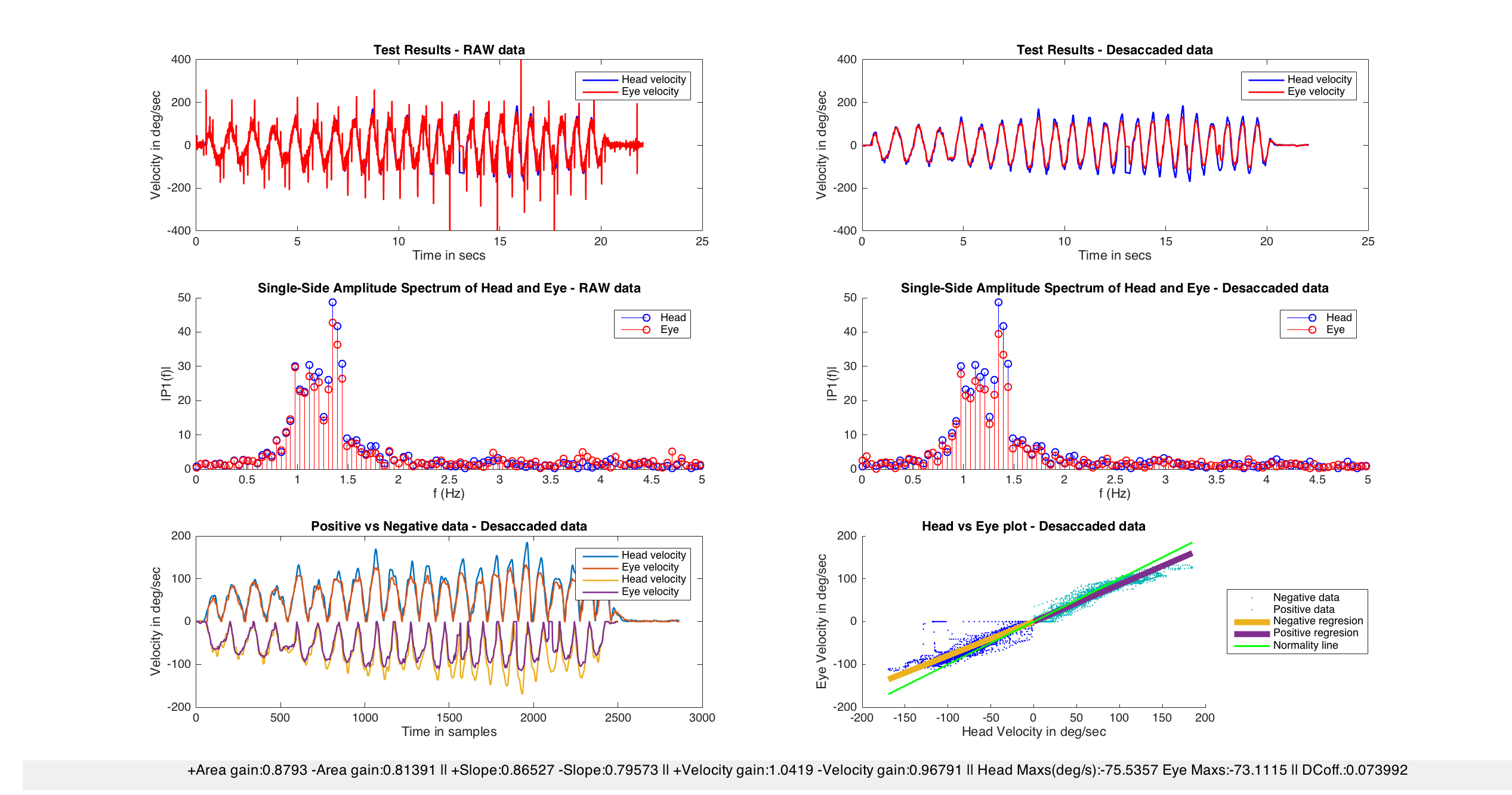

Supplement: Supplementary file 1 [file data_sheet_1.ZIP › RESULTS/PARTICIPANTS_TEST/B5.png]

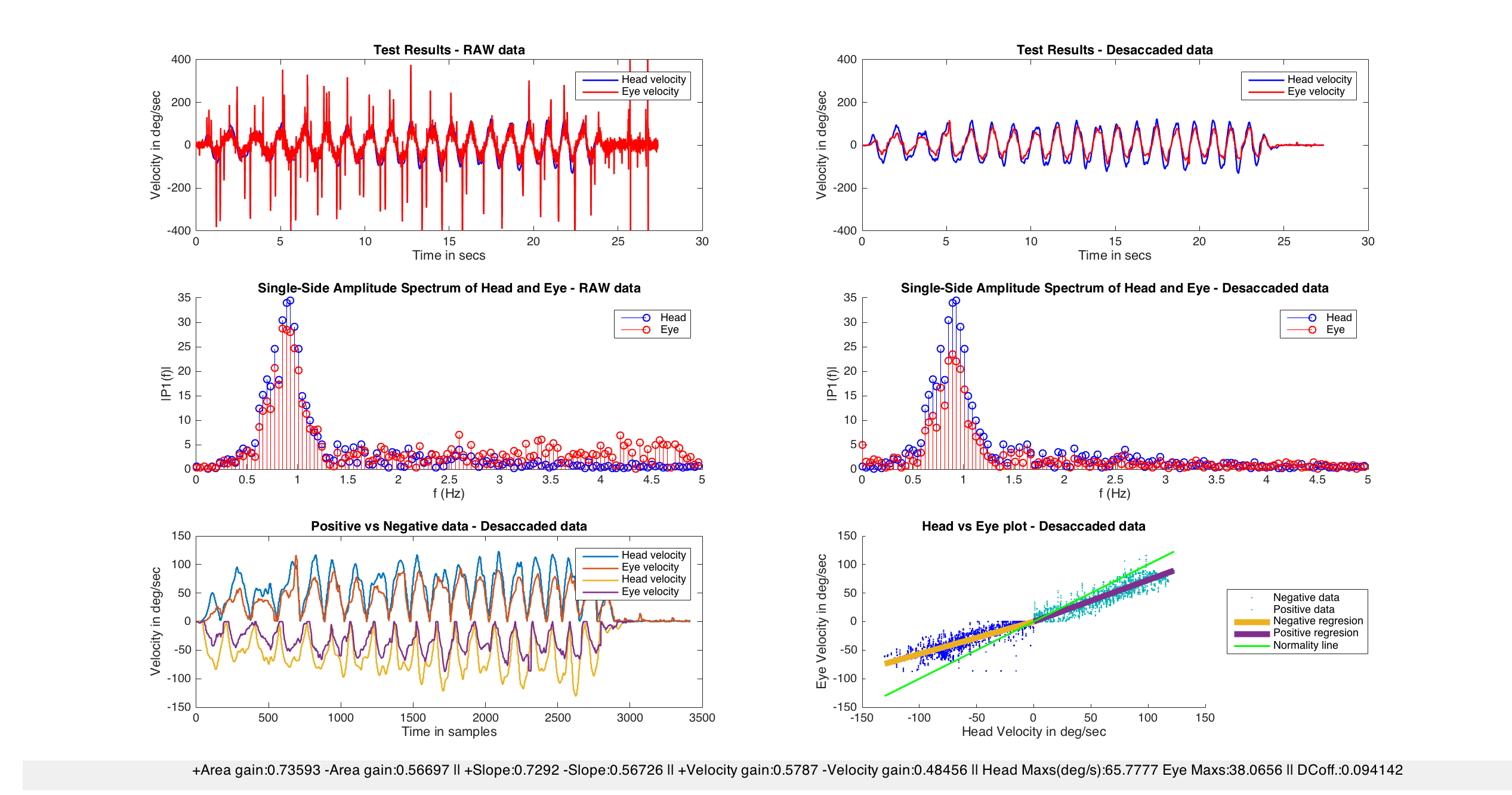

Supplement: Supplementary file 1 [file data_sheet_1.ZIP › RESULTS/PARTICIPANTS_TEST/B6.png]

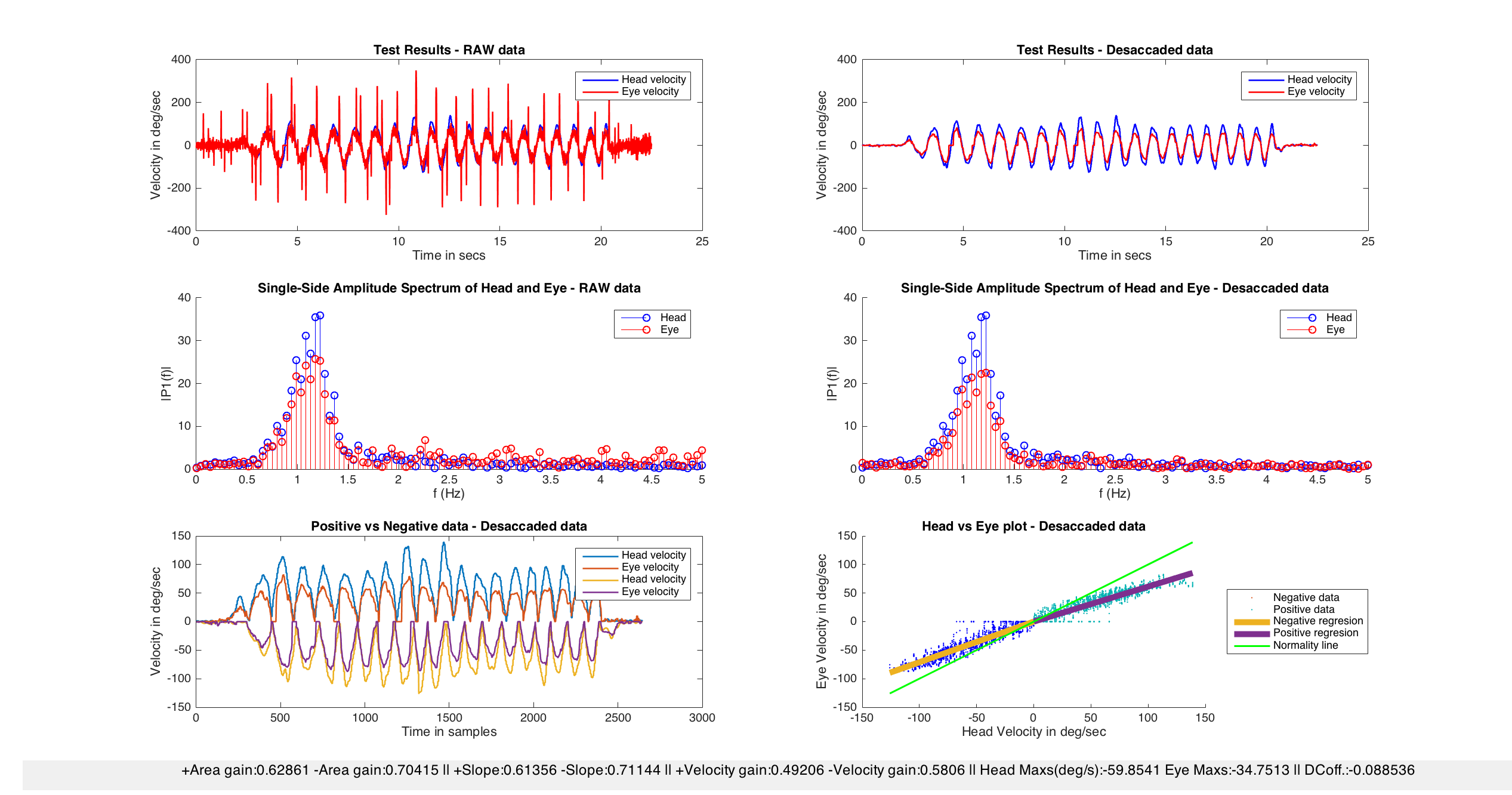

Supplement: Supplementary file 1 [file data_sheet_1.ZIP › RESULTS/PARTICIPANTS_TEST/B7.png]

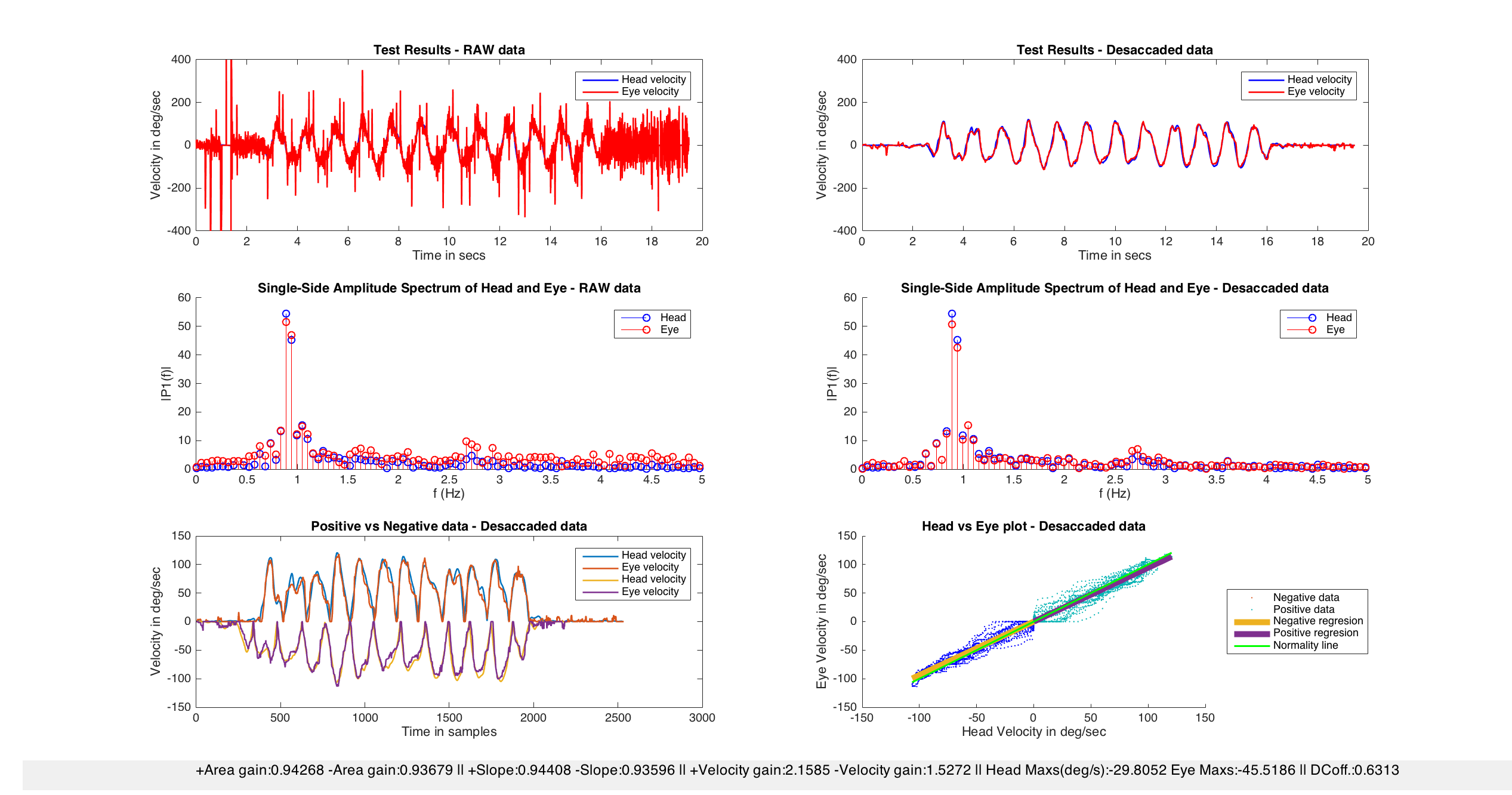

Supplement: Supplementary file 1 [file data_sheet_1.ZIP › RESULTS/PARTICIPANTS_TEST/B8.png]

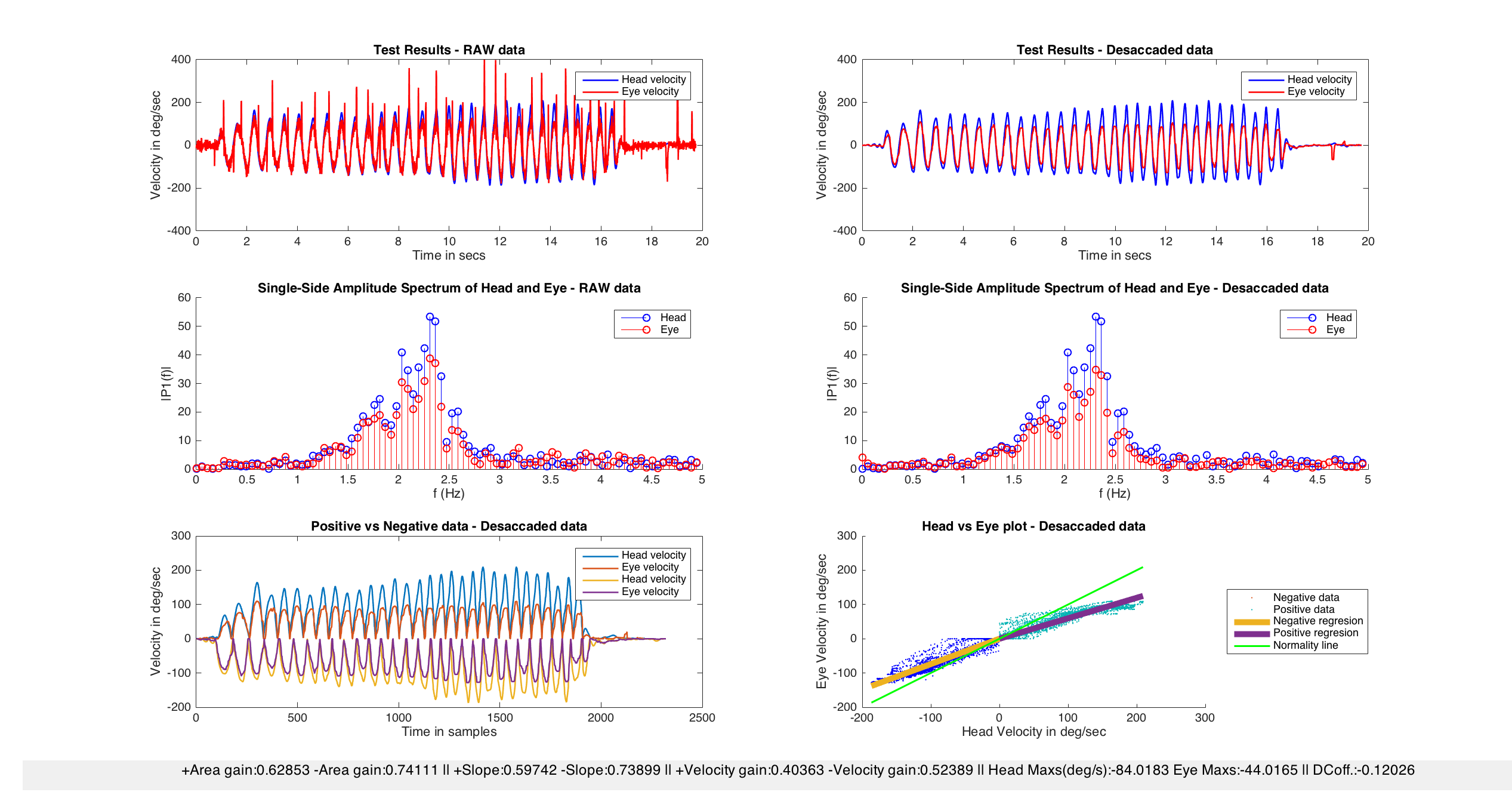

Supplement: Supplementary file 1 [file data_sheet_1.ZIP › RESULTS/PARTICIPANTS_TEST/B9.png]

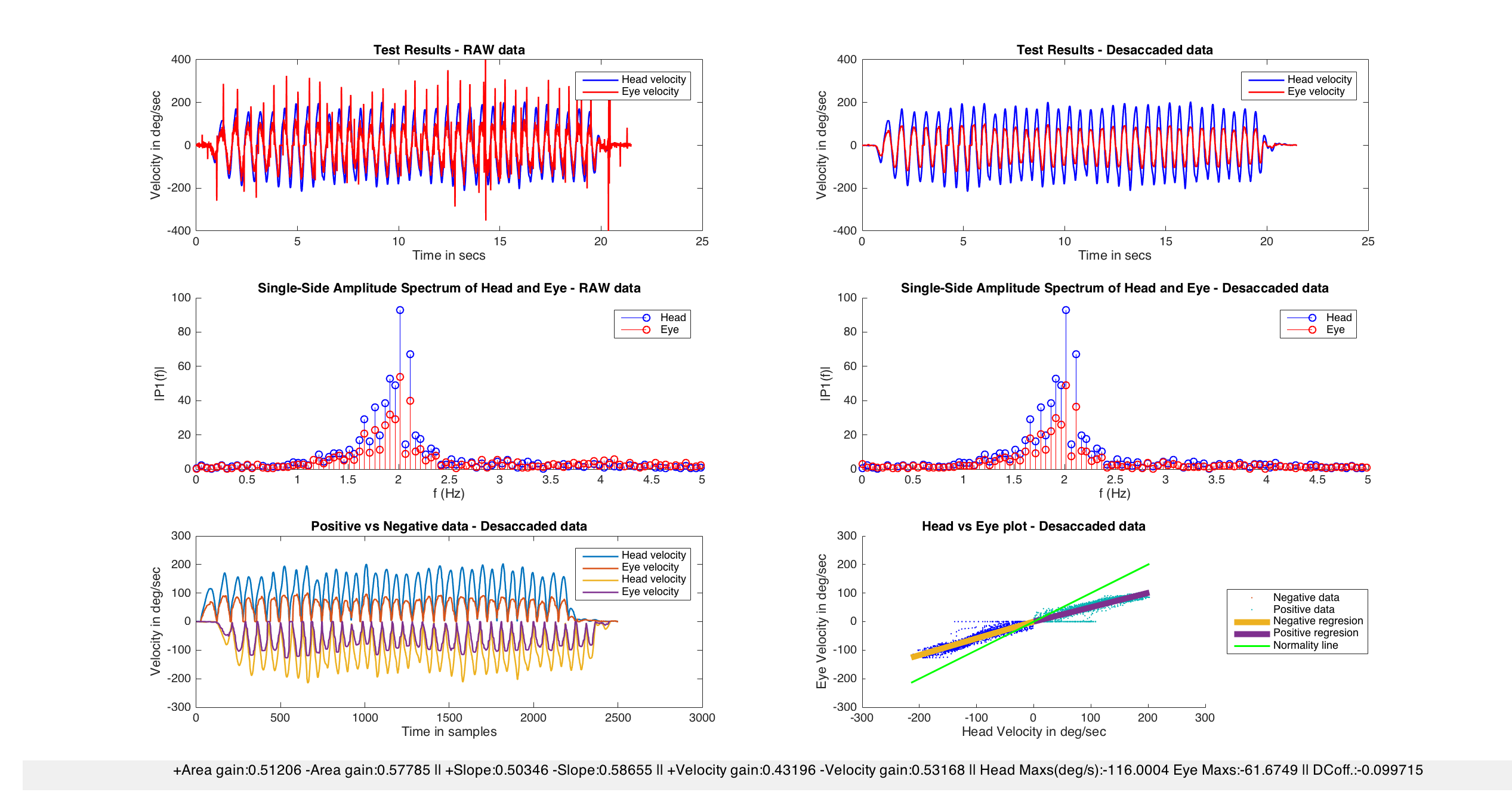

Supplement: Supplementary file 1 [file data_sheet_1.ZIP › RESULTS/PARTICIPANTS_TEST/C1.png]

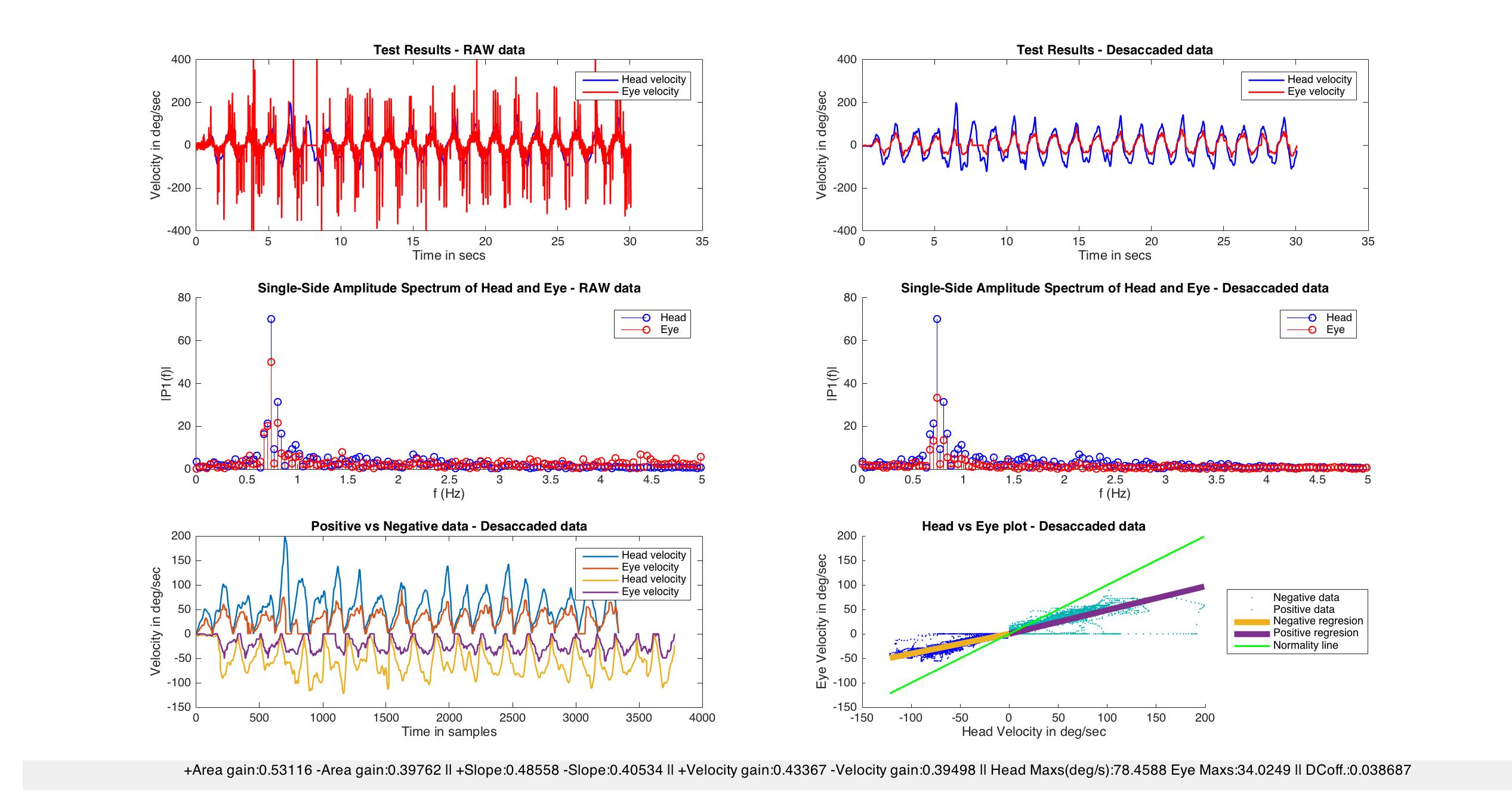

Supplement: Supplementary file 1 [file data_sheet_1.ZIP › RESULTS/PARTICIPANTS_TEST/C2.png]

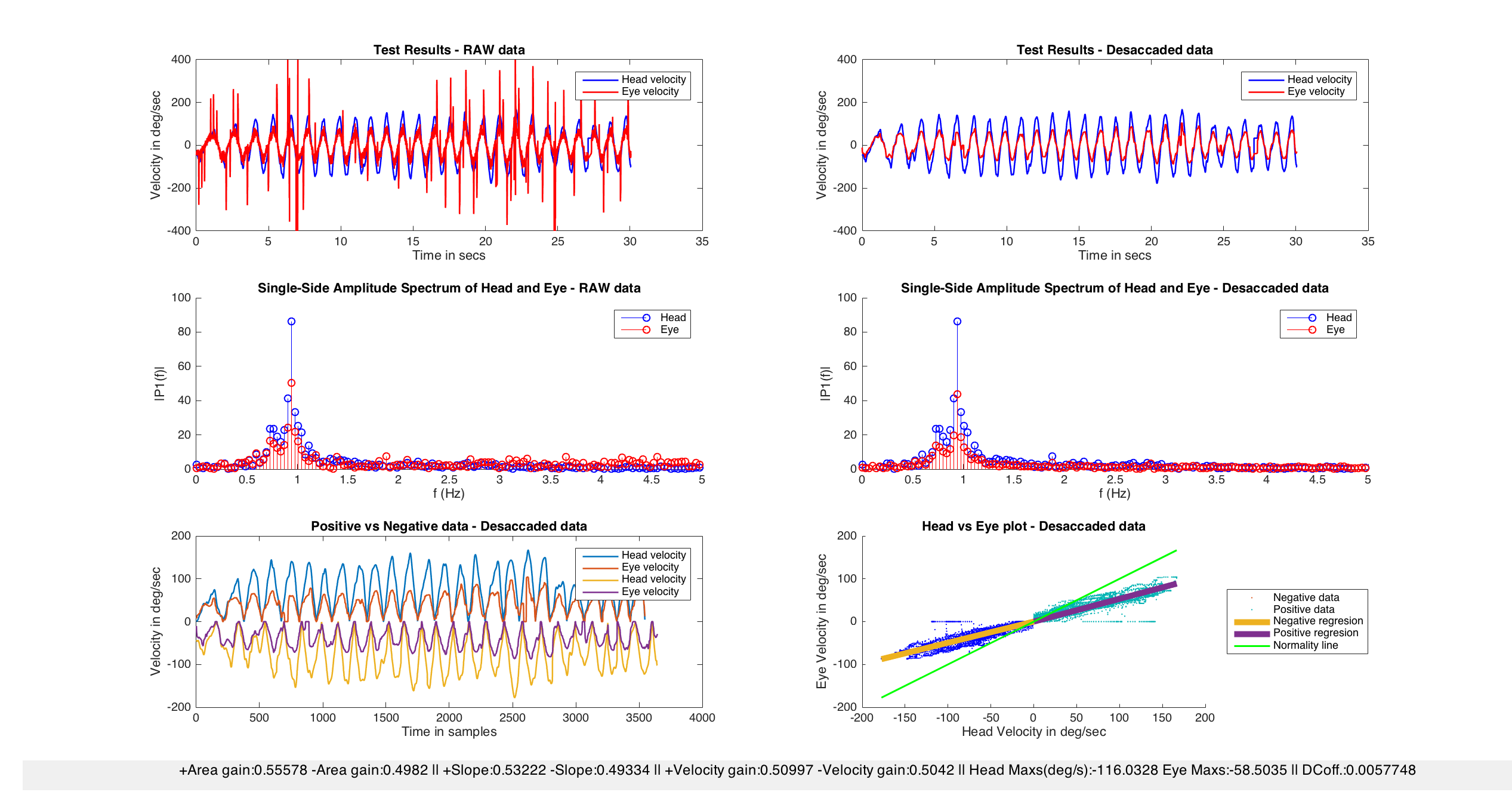

Supplement: Supplementary file 1 [file data_sheet_1.ZIP › RESULTS/PARTICIPANTS_TEST/C3.png]

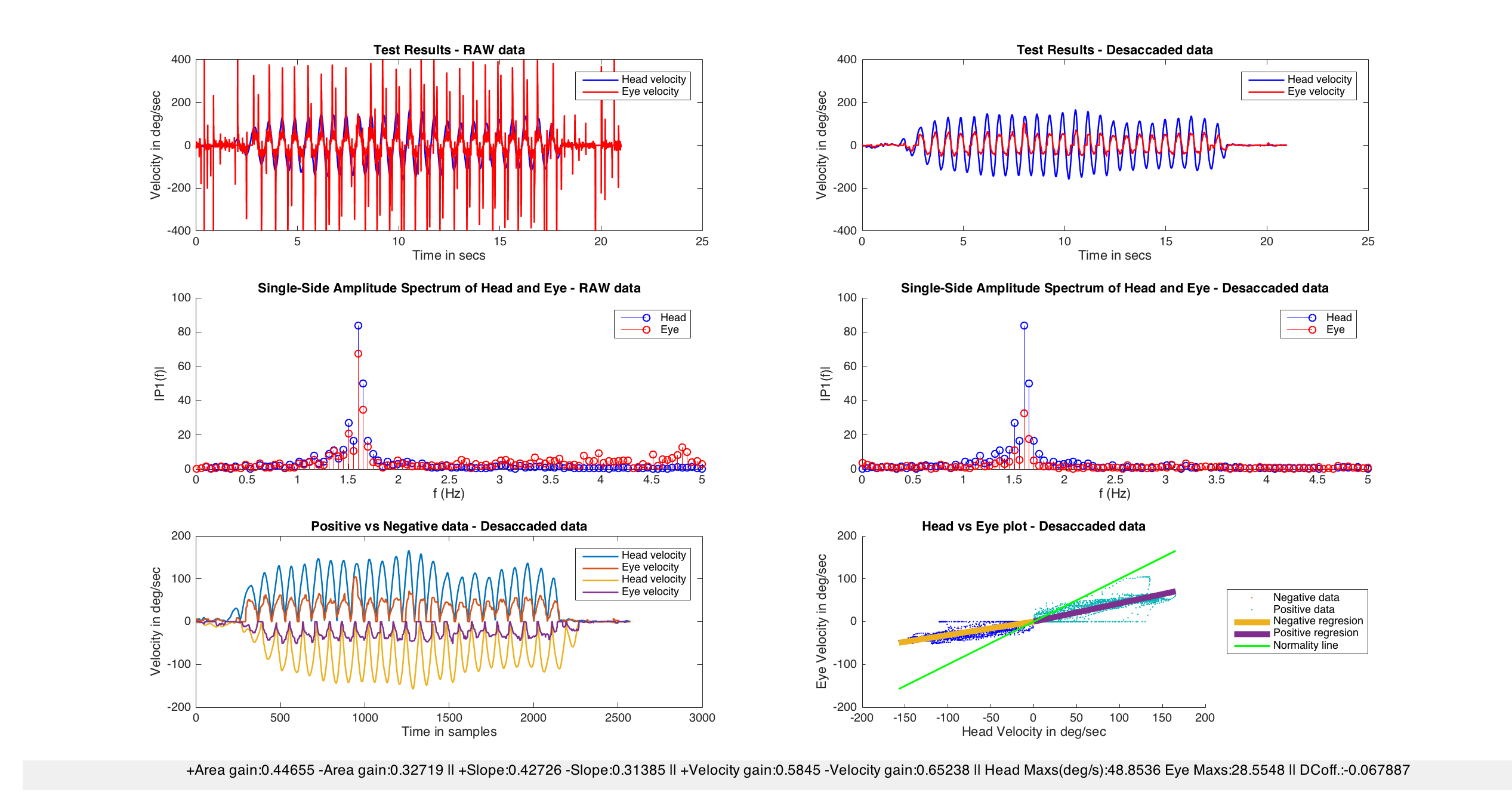

Supplement: Supplementary file 1 [file data_sheet_1.ZIP › RESULTS/PARTICIPANTS_TEST/C4.png]

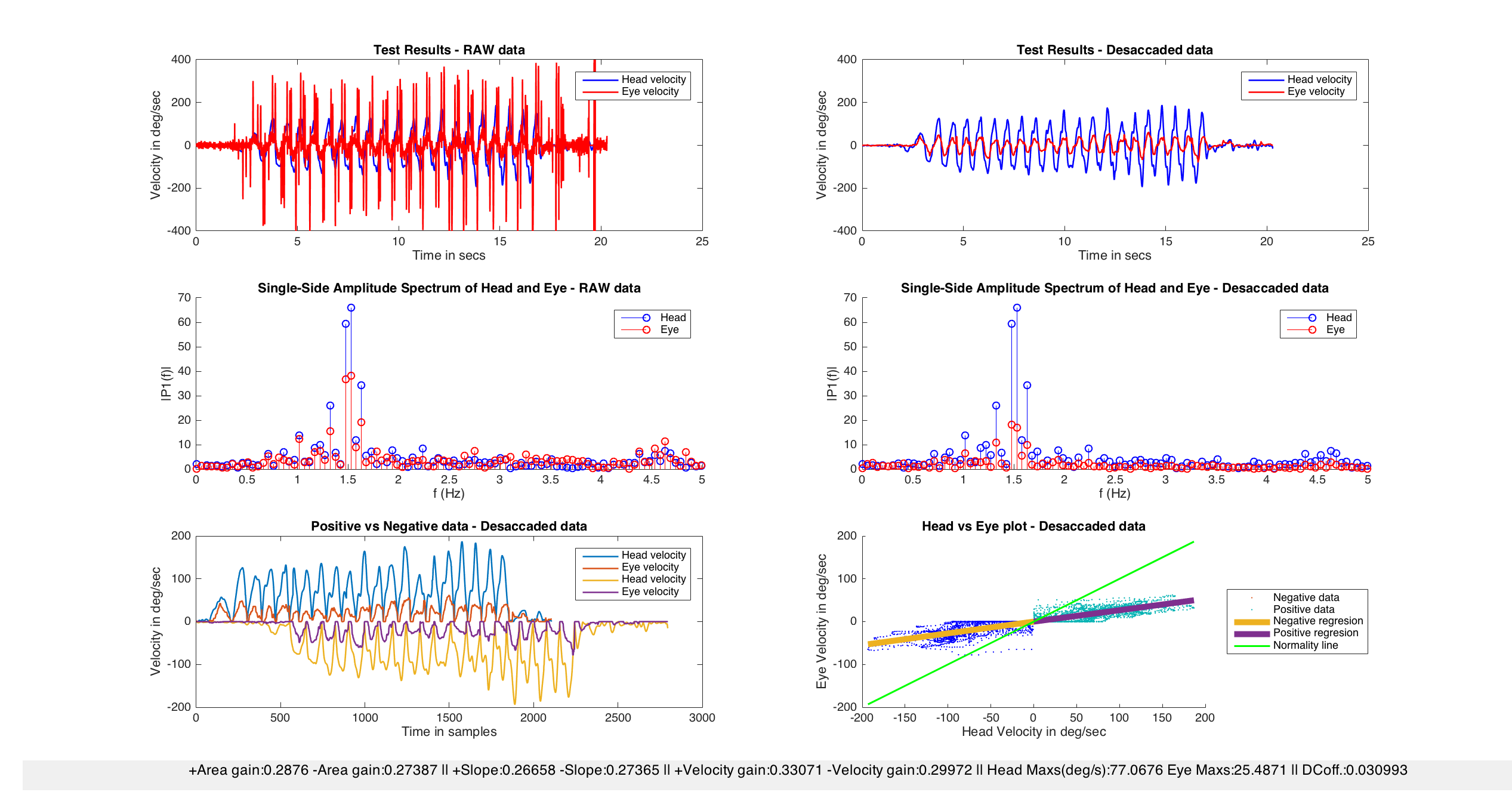

Supplement: Supplementary file 1 [file data_sheet_1.ZIP › RESULTS/PARTICIPANTS_TEST/C5.png]

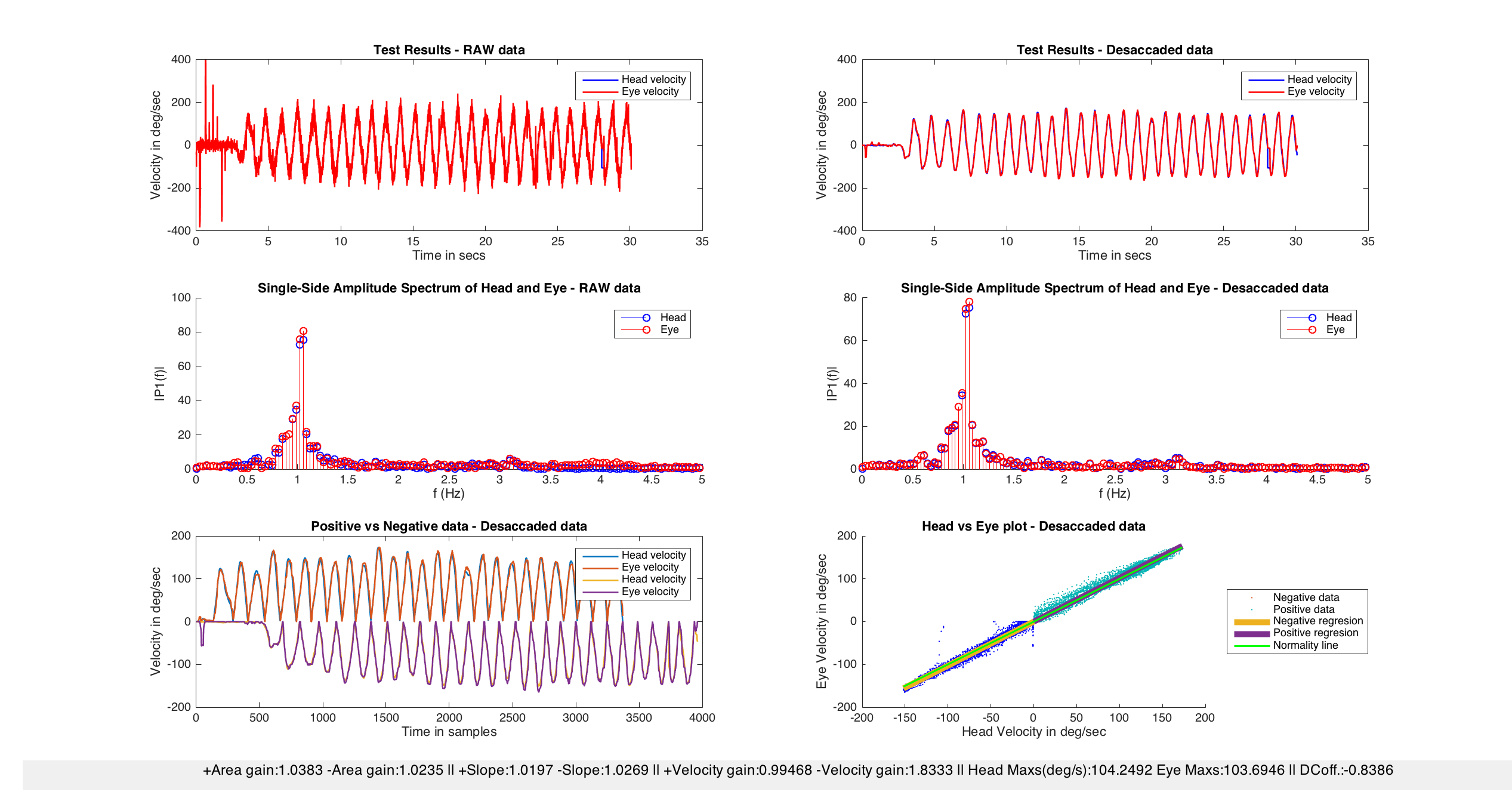

Supplement: Supplementary file 1 [file data_sheet_1.ZIP › RESULTS/PARTICIPANTS_TEST/E1.png]

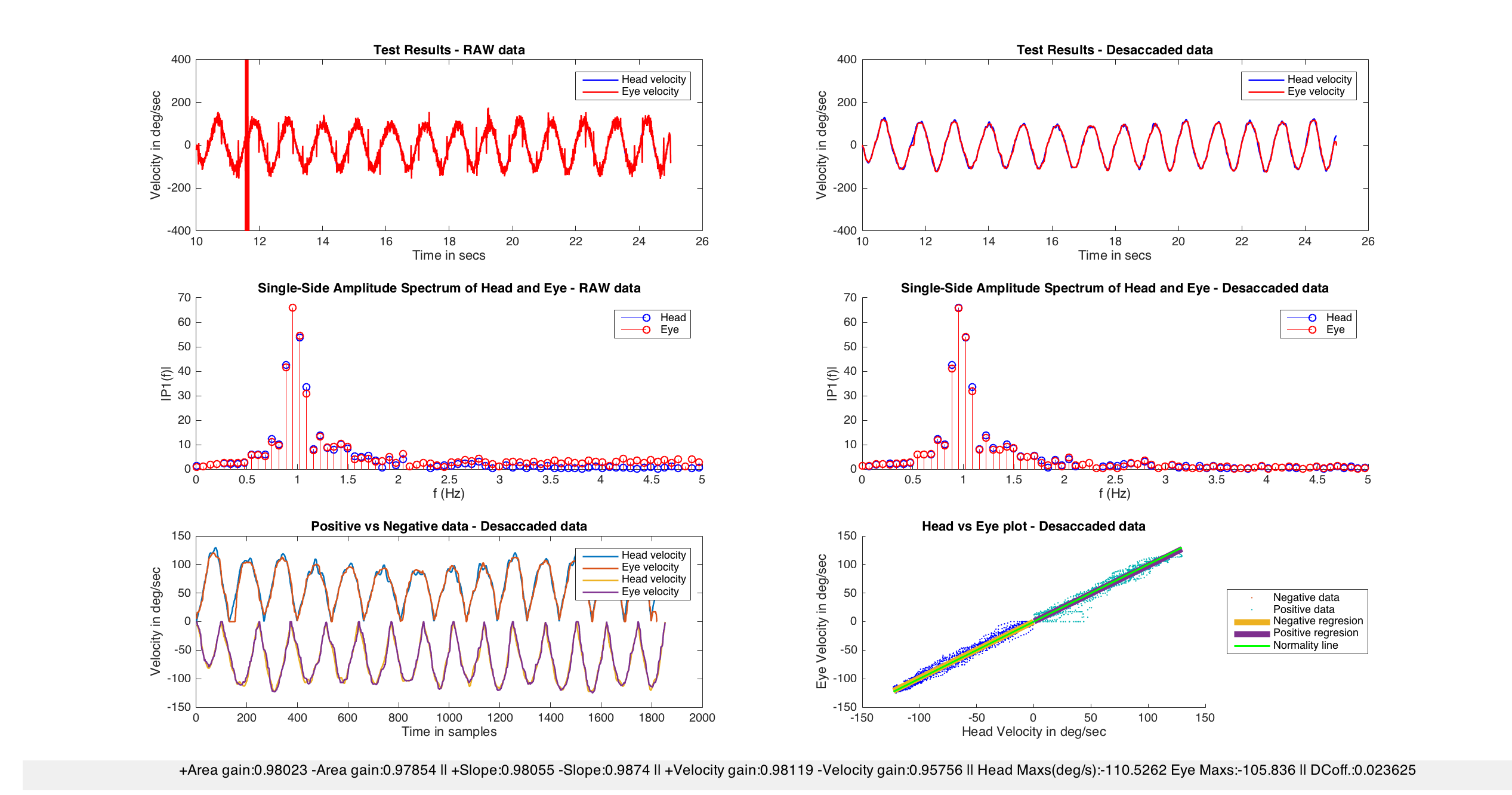

Supplement: Supplementary file 1 [file data_sheet_1.ZIP › RESULTS/PARTICIPANTS_TEST/E10.png]

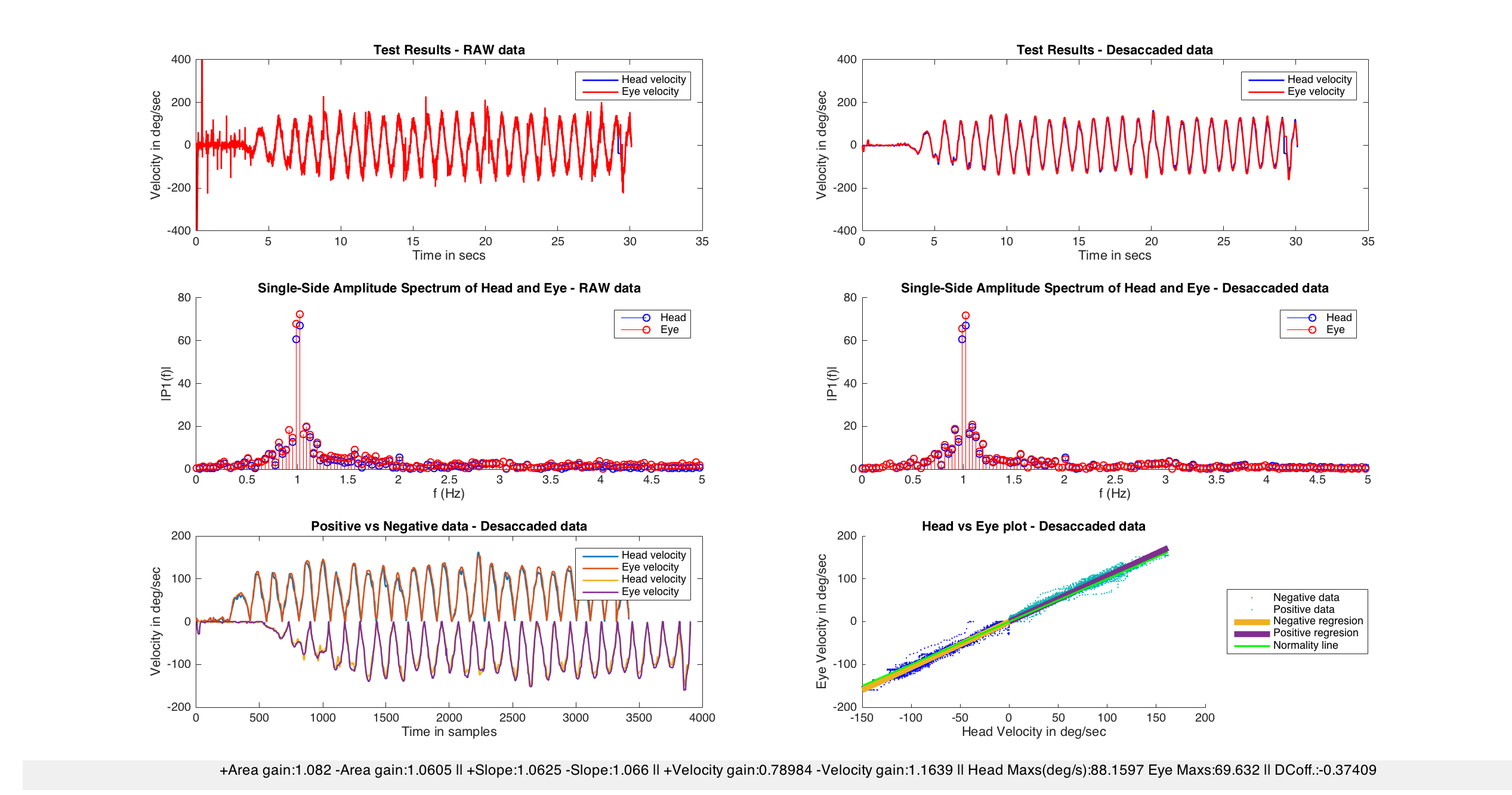

Supplement: Supplementary file 1 [file data_sheet_1.ZIP › RESULTS/PARTICIPANTS_TEST/E2.png]

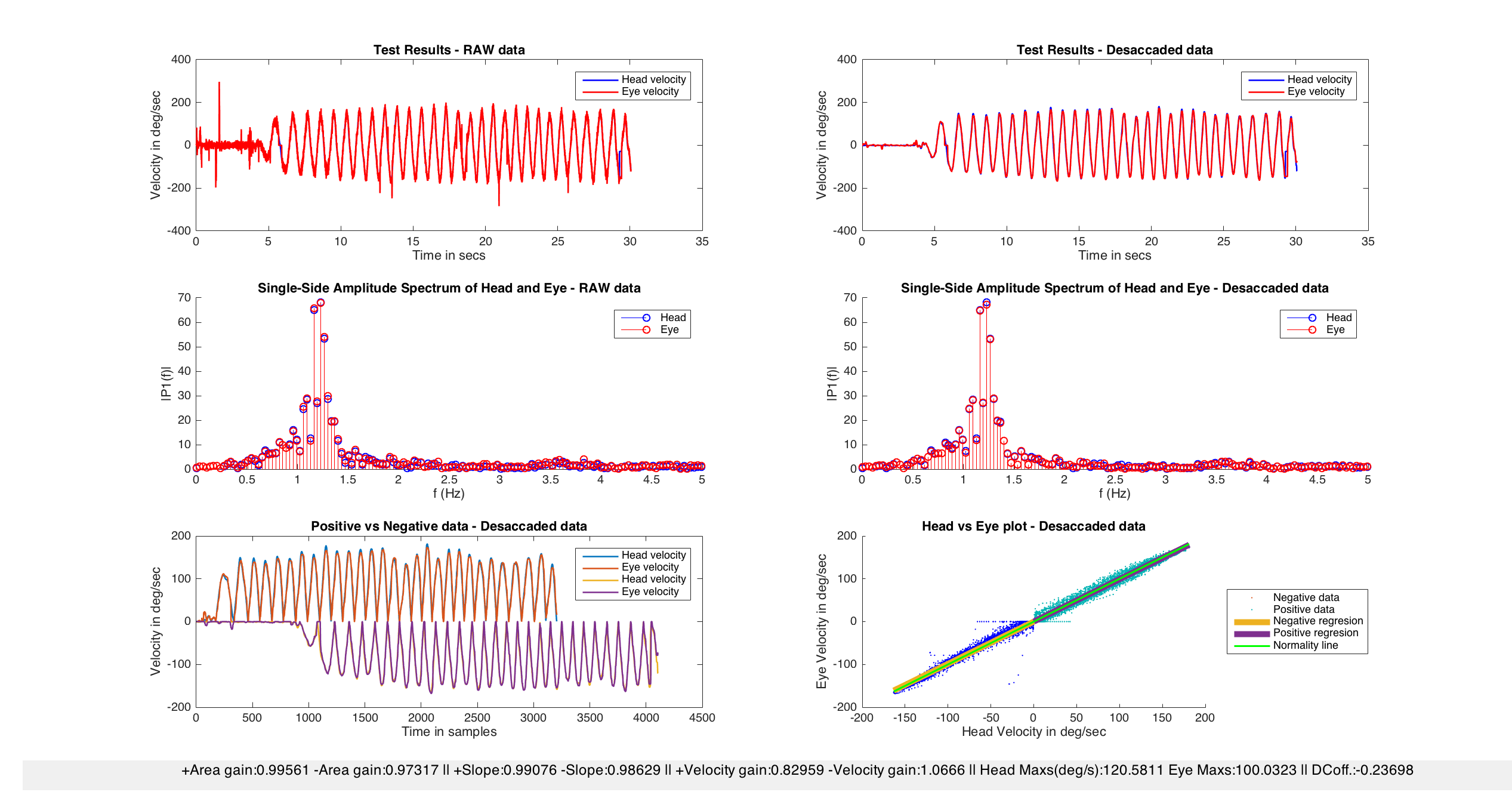

Supplement: Supplementary file 1 [file data_sheet_1.ZIP › RESULTS/PARTICIPANTS_TEST/E3.png]

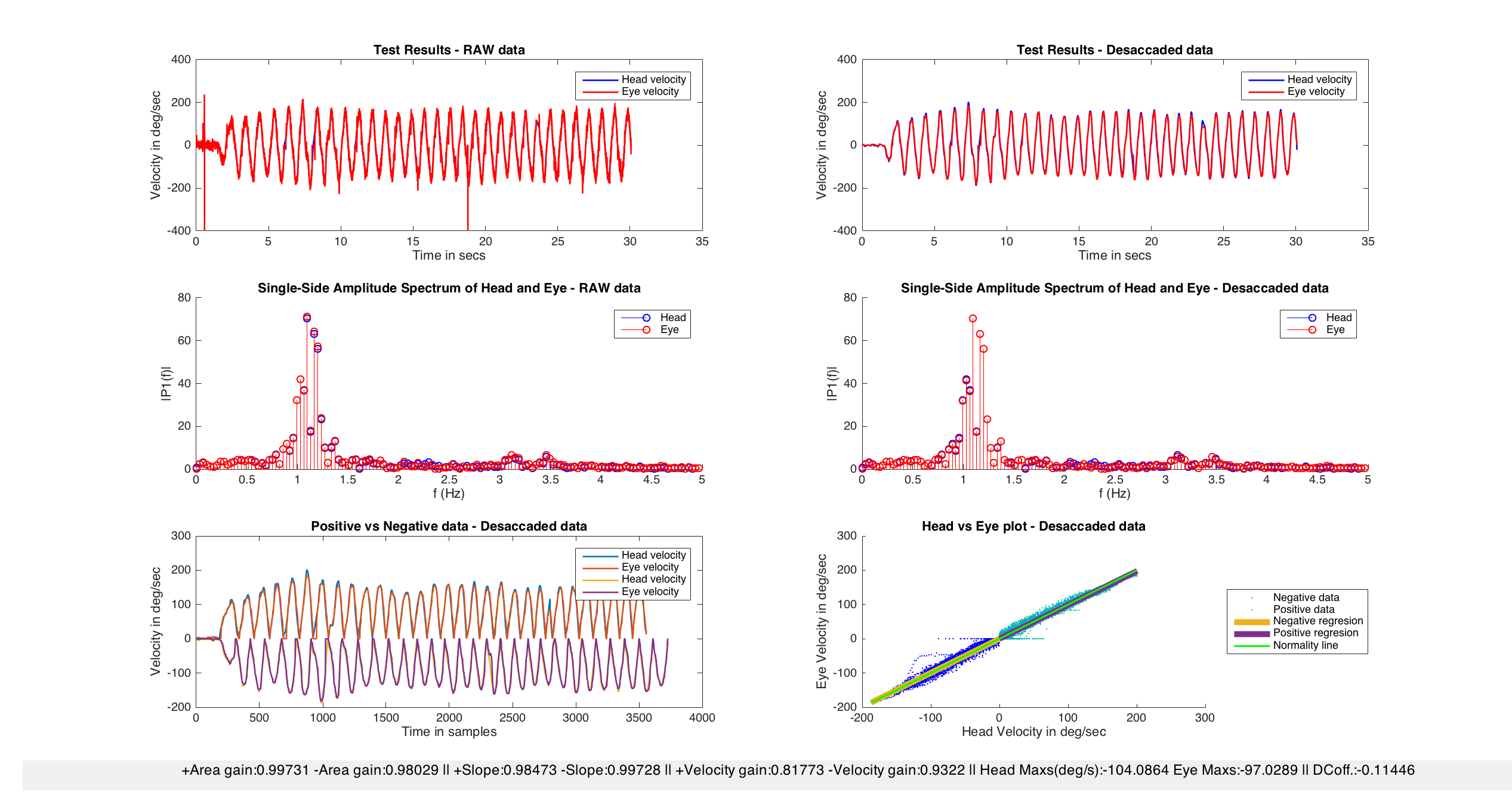

Supplement: Supplementary file 1 [file data_sheet_1.ZIP › RESULTS/PARTICIPANTS_TEST/E4.png]

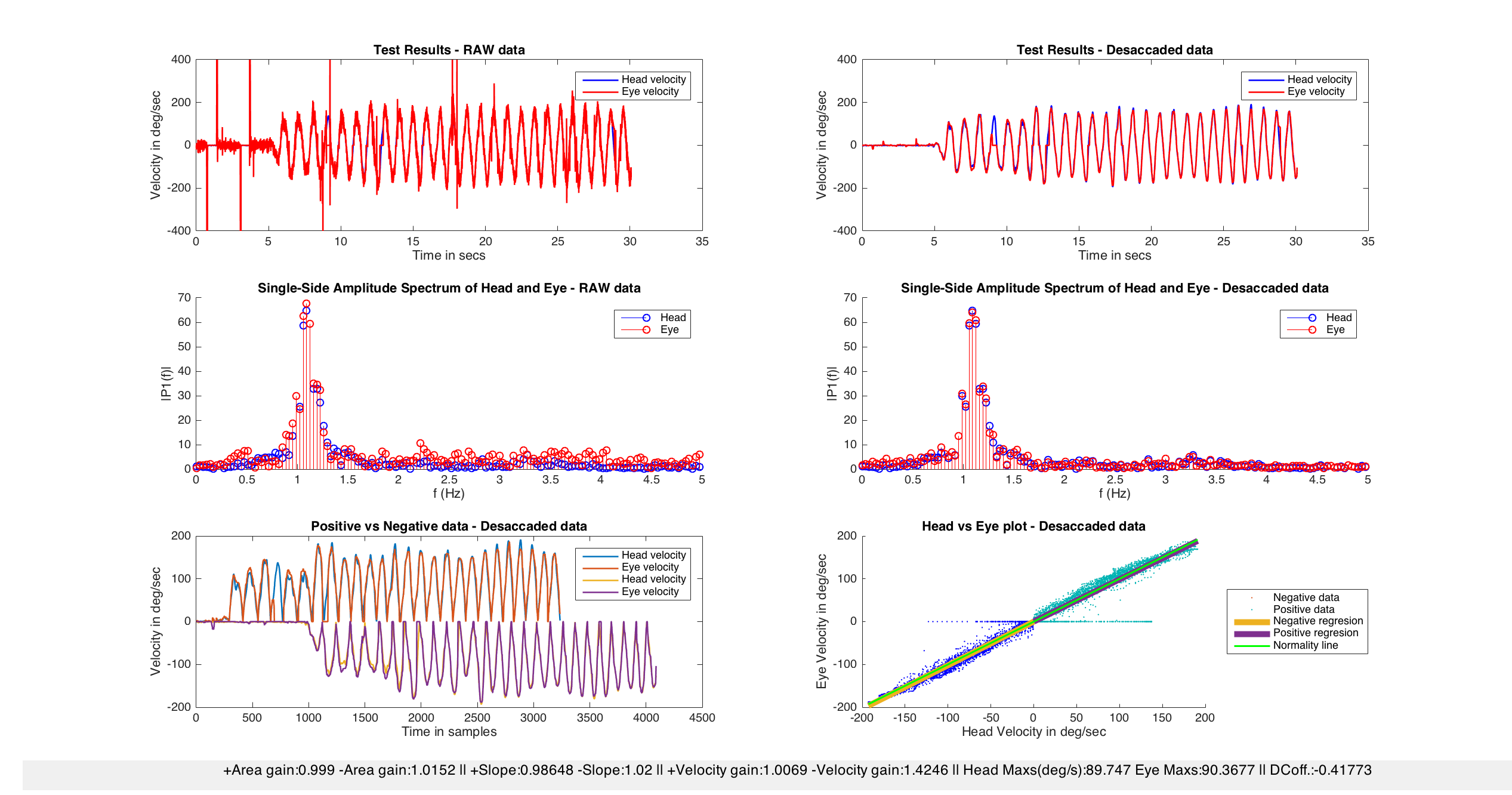

Supplement: Supplementary file 1 [file data_sheet_1.ZIP › RESULTS/PARTICIPANTS_TEST/E5.png]

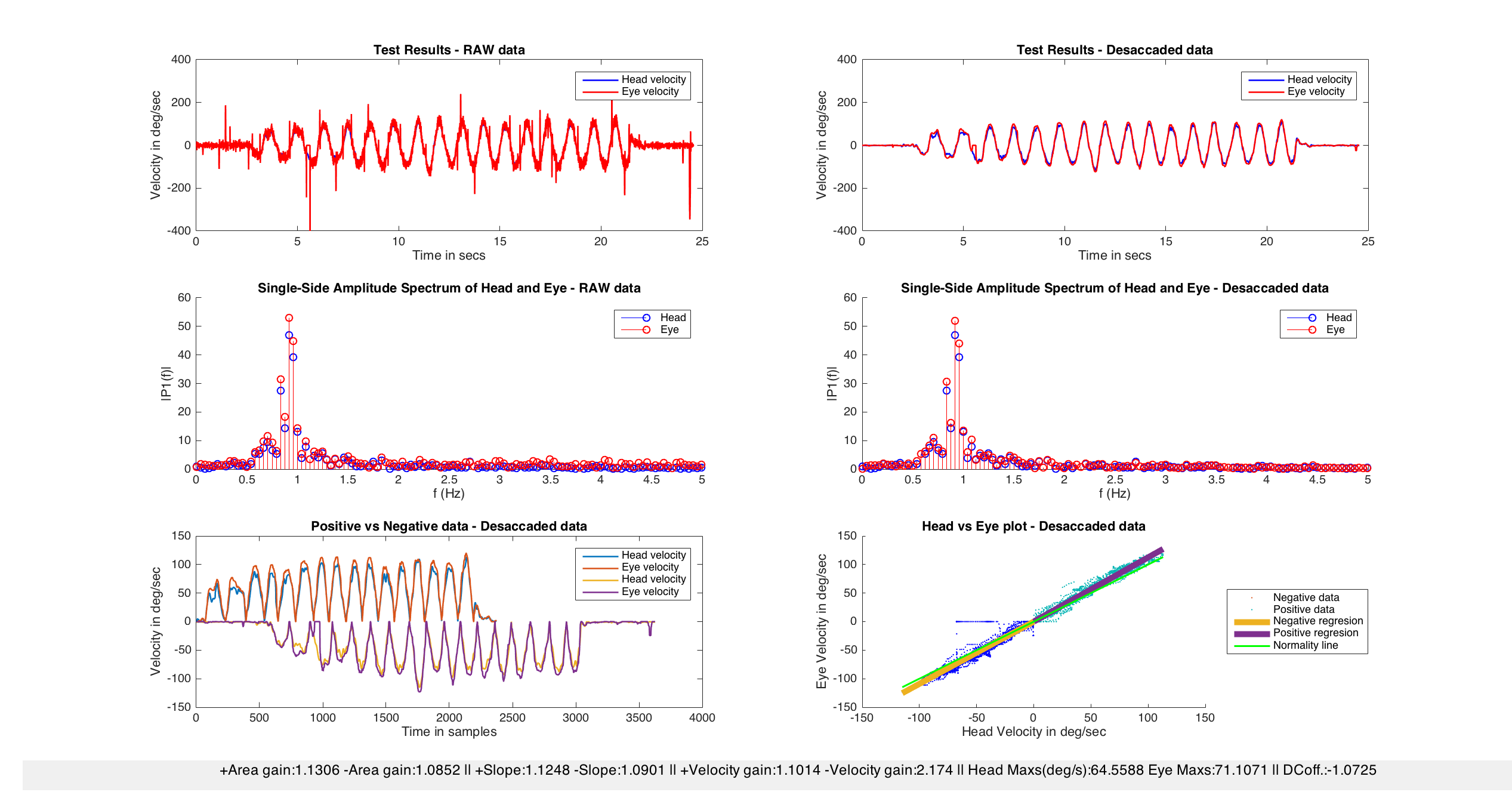

Supplement: Supplementary file 1 [file data_sheet_1.ZIP › RESULTS/PARTICIPANTS_TEST/E6.png]

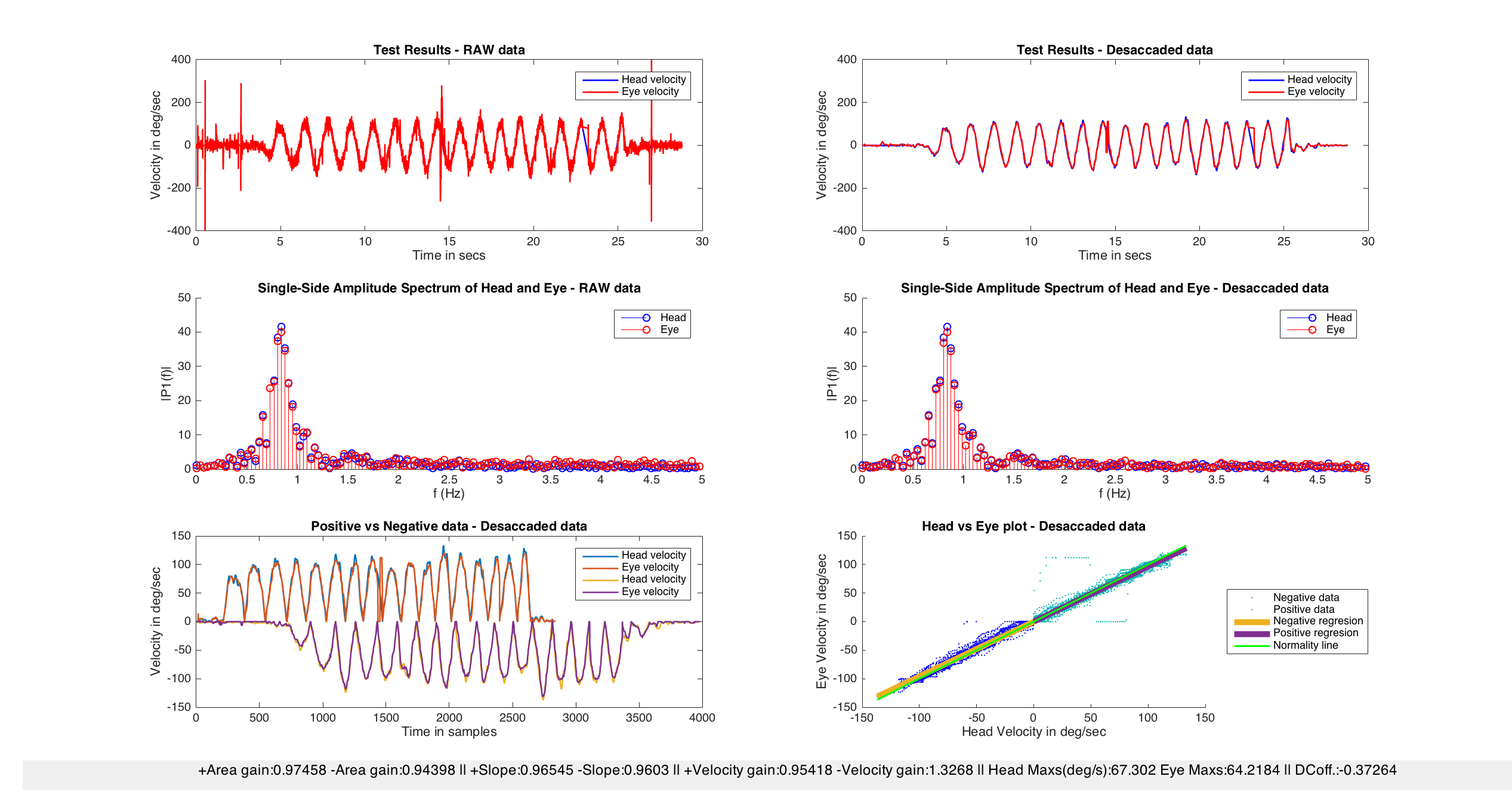

Supplement: Supplementary file 1 [file data_sheet_1.ZIP › RESULTS/PARTICIPANTS_TEST/E7.png]

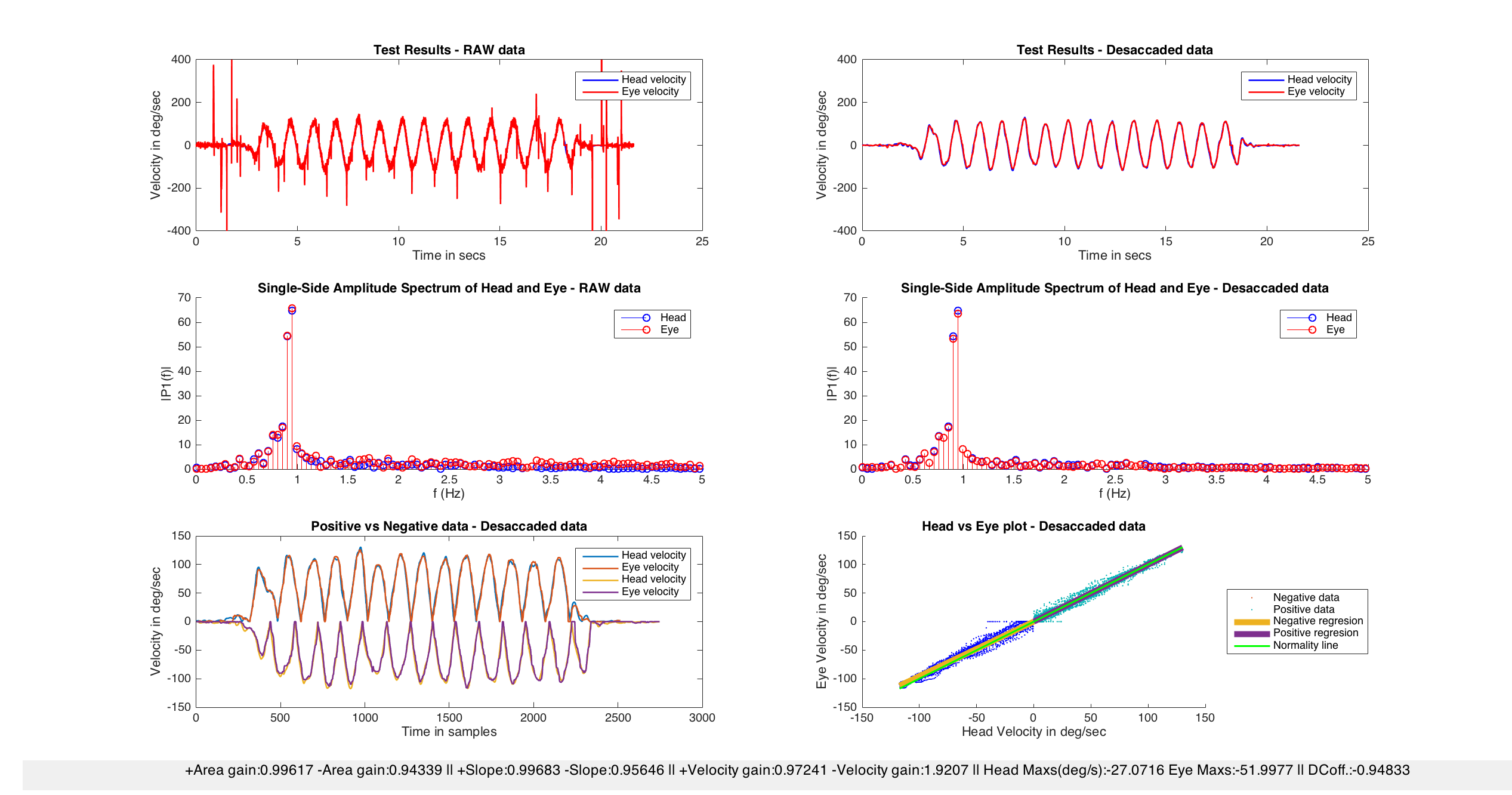

Supplement: Supplementary file 1 [file data_sheet_1.ZIP › RESULTS/PARTICIPANTS_TEST/E8.png]

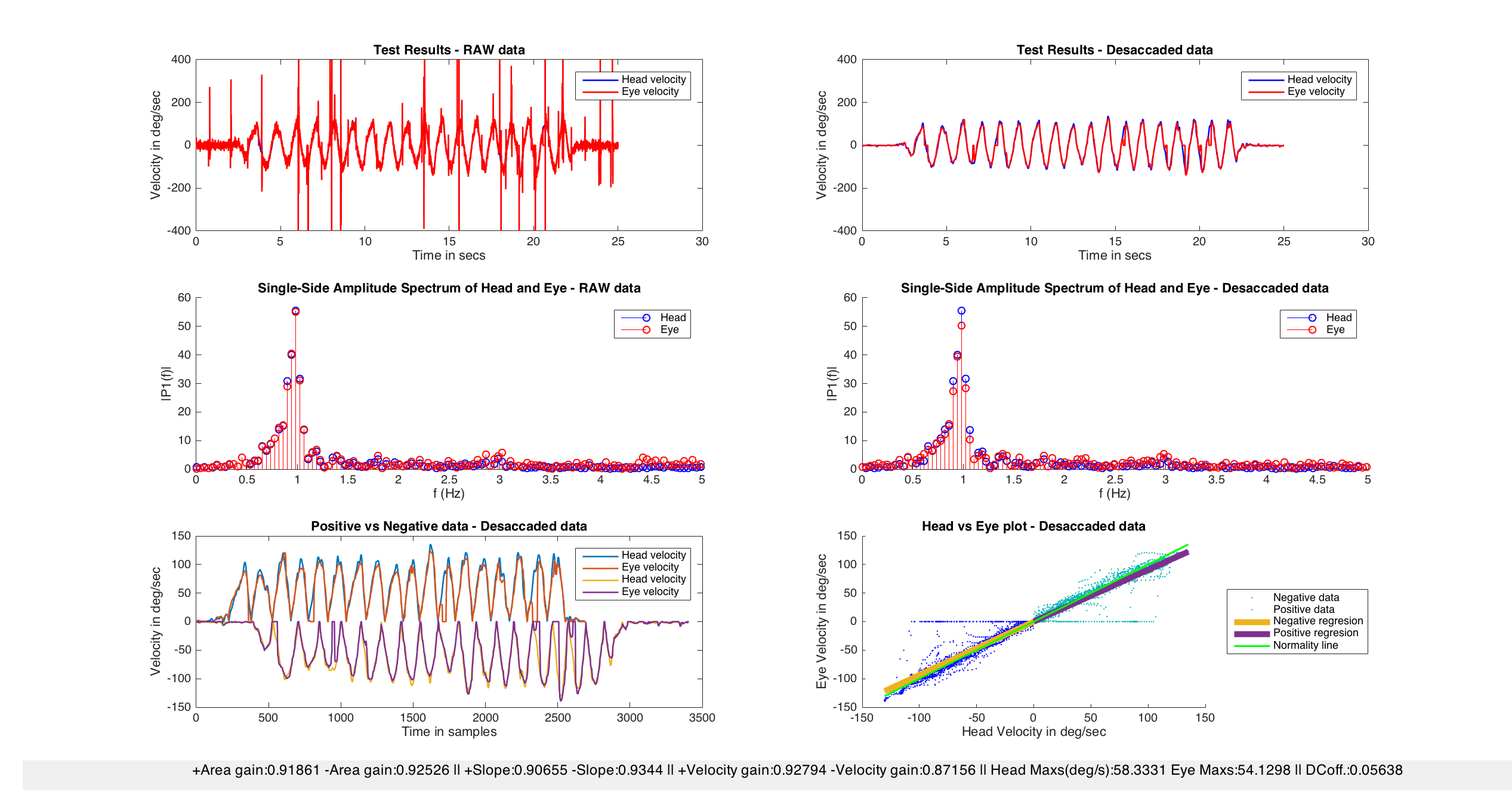

Supplement: Supplementary file 1 [file data_sheet_1.ZIP › RESULTS/PARTICIPANTS_TEST/E9.png]
